# Autoimmune diseases and their genetic link to bronchiectasis: insights from a genetic correlation and Mendelian randomization study

**DOI:** 10.3389/fimmu.2024.1343480

**Published:** 2024-04-10

**Authors:** Yue Su, Youqian Zhang, Yanhua Chai, Jinfu Xu

**Affiliations:** ^1^ Department of Respiratory and Critical Care Medicine, Shanghai Pulmonary Hospital, School of Medicine, Tongji University, Shanghai, China; ^2^ Health Science Center, Yangtze University, Jingzhou, Hubei, China

**Keywords:** autoimmune diseases, bronchiectasis, rheumatoid arthritis, Mendelian randomization, Crohn’s disease

## Abstract

**Background:**

Previous studies have demonstrated that autoimmune diseases are closely associated with bronchiectasis (BE). However, the causal effects between autoimmune diseases and BE remain elusive.

**Methods:**

All summary-level data were obtained from large-scale Genome-Wide Association Studies (GWAS). The univariate Mendelian randomization (UVMR) was utilized to investigate the genetic causal correlation (r_g_) of 12 autoimmune diseases and bronchiectasis, The Multivariable Mendelian Randomization (MVMR) method was used to explore the effects of the confounding factors. Further investigation was conducted to identify potential intermediate factors using mediation analysis. Finally, the linkage disequilibrium score regression (LDSC) method was used to identify genetic correlations among complex traits. A series of sensitivity analyses was performed to validate the robustness of the results.

**Results:**

The LDSC analysis revealed significant genetic correlations between BE and Crohn’s disease (CD) (r_g_ = 0.220, *P* = 0.037), rheumatoid arthritis (RA) (r_g_ = 0.210, *P* = 0.021), and ulcerative colitis (UC) (r_g_ = 0.247, *P* = 0.023). However, no genetic correlation was found with other autoimmune diseases (*P* > 0.05). The results of the primary IVW analysis suggested that for every SD increase in RA, there was a 10.3% increase in the incidence of BE (odds ratio [OR] = 1.103, 95% confidence interval [CI] 1.055-1.154, *P* = 1.75×10^-5^, FDR = 5.25×10^-5^). Furthermore, for every standard deviation (SD) increase in celiac disease (CeD), the incidence of BE reduced by 5.1% (OR = 0.949, 95% CI 0.902-0.999, *P* = 0.044, FDR = 0.044). We also observed suggestive evidence corresponding to a 3% increase in BE incidence with T1DM (OR = 1.033, 95% CI 1.001-1.066, *P* = 0.042, FDR = 0.063). Furthermore, MVMR analysis showed that RA was an independent risk factor for BE, whereas mediator MR analysis did not identify any mediating factors. The sensitivity analyses corroborated the robustness of these findings.

**Conclusion:**

LDSC analysis revealed significant genetic correlations between several autoimmune diseases and BE, and further MVMR analysis showed that RA is an independent risk factor for BE.

## Introduction

Bronchiectasis (BE) is a chronic respiratory disease characterized by the clinical symptoms of cough, sputum production, and hemoptysis in the presence of abnormal, irreversible dilatation of the bronchi that can be diagnosed using high-resolution chest computed tomography (CT) (1, 2). There has been a marked increase in the overall prevalence of bronchiectasis worldwide. In China, the prevalence of bronchiectasis increased 2.31-fold between 2013 and 2017, from 75.48 to 174.45 per 100,000 (3). Moreover, the prevalence of BE in females is higher than in males and also increases with age (4, 5). Importantly, BE is a heterogeneous syndrome caused by several underlying factors, such as pulmonary infections, cystic fibrosis (CF), primary ciliary dyskinesia (PCD), immunodeficiency disorders, allergic bronchopulmonary aspergillosis (ABPA), and autoimmune diseases. Recently, the association between BE and autoimmune diseases has been well recognized, and available studies have suggested that the oral, lung, and gut microbiota may affect the autoimmunity and structural integrity of the airways that contribute to BE (6). Neel et al. suggested that BE is highly prevalent in anti-myeloperoxidase (MPO) antineutrophil cytoplasmic autoantibody (ANCA)-associated vasculitis, and anti-MPO patients with BE have a higher risk of peripheral neuropathy (7). A systematic review and meta-analysis by Martin et al. demonstrated that BE may be a common extra-articular manifestation of rheumatoid arthritis (RA) (8), and anti-cyclic citrullinated peptide (CCP) antibodies (ACPAs) are associated with more severe RA-BE. However, the causal effects between BE and autoimmune diseases remain unclear.

Mendelian Randomization (MR) represents a methodological approach employing genetic variants as instrumental variables (IVs) sourced from genome-wide association studies (GWAS) to evaluate the causal relationship between a risk factor (exposure) and a resultant outcome (9). Contrary to traditional observational analyses, MR offers a more accurate estimation of the causal effect by considerably reducing the impact of confounders (10). The linkage disequilibrium score (LDSC) regression serves as a tool for estimating trait heritability, reflecting the percentage of trait variance ascribed to genetic determinants. Furthermore, LDSC assesses the genetic correlation between various traits using GWAS-derived summary statistics (11, 12). The objective of this research was to explore the plausible causal linkage between BE and autoimmune disorders.

## Materials and methods

### Study design

The foundational data for this investigation was retrieved from publicly available summary-level datasets from GWAS. Univariate Mendelian Randomization (UVMR), Multivariable Mendelian Randomization (MVMR), genetic correlation, and colocalization analyses were used to elucidate the causal interplay between autoimmune disorders and outcome phenotypes.

**Figure 1:**
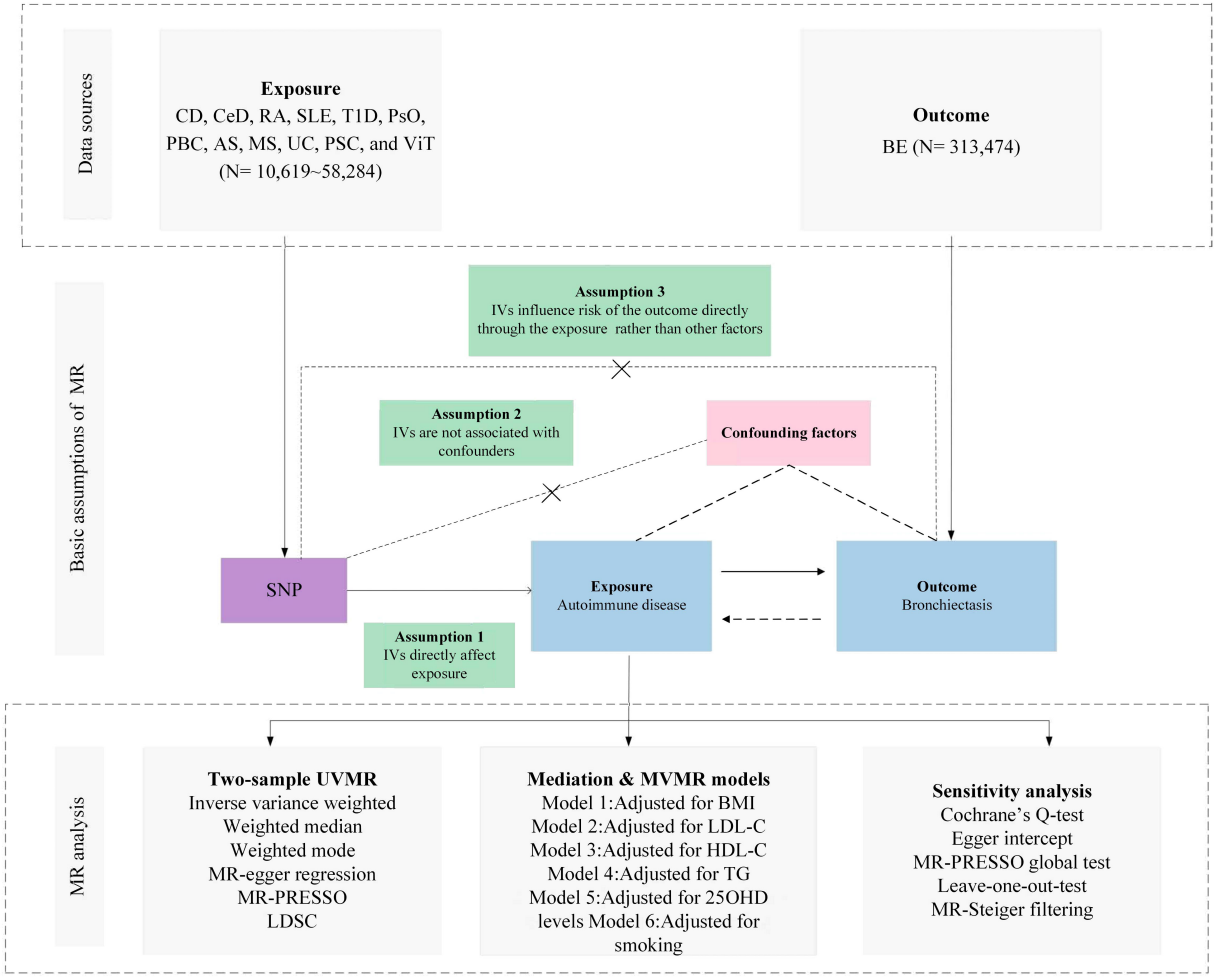
Overview of research design and analysis strategy. Overview of the research design. The MR framework is based on three fundamental MR assumptions, with MVMR analyses adjusting for six mediating factors for positive results. MR, Mendelian Randomization; MVMR: Multivariate Mendelian Randomization; UVMR, Univariate Mendelian Randomization; BMI, Body Mass Index; SNP, Single Nucleotide Polymorphism; MR- PRESSO, MR Pleiotropy Residual Sum and Outlier; LDL-C, Low Density Lipoprotein Cholesterol; HDL-C, High Density Lipoprotein Cholesterol; TG, Triglyceride; 25OHD, 25-hydroxyvitamin D; CD, Crohn's disease; CeD, Celiac disease; MS, Multiple sclerosis; RA, Rheumatoid arthritis; SLE, Systemic lupus erythematosus; UC, Ulcerative colitis; TID, Type 1 diabetes; PsO, Psoriasis; PSC, Primary sclerosing cholangitis; PBC, Primary biliary cirrhosis; AS, Ankylosing spondylitis; VIT, Vitiligo; BE, Bronchiectasis; LDSC, linkage disequilibrium score regression.

The selection of Instrumental Variables (IVs) for exposure was grounded in a tripartite criterion: i) the nominated genetic determinant, earmarked as the instrumental variable, must display a robust affiliation with the exposure; ii) the genetic determinant must not be intertwined with any potential confounders; and iii) the influence of genetic determinants on the outcome is channeled exclusively through its interaction with the exposure, thus eliminating the prospect of secondary routes (13). The architectural blueprint of the MR is illustrated in **Figure 1** and **Table 1**, along with **Supplementary File 1**, which provides a comprehensive exposition of the summary statistics data repositories.

**Table 1:** Detailed information of data sources.

Explore or Outcome	Ref	Consortium	Ancestry	Participants
Phenotypes				
CD	28067908	de Lange KM et al	European	12,194 cases and 28,072 controls
CeD	22057235	Trynka et al	European	12,041 cases and 12,228 controls
MS	24076602	IMSGC	European	14,498 cases and 24,091 controls
RA	33310728	Ha E et al	European	14,361 cases and 43,923 controls
SLE	26502338	Bentham J et al	European	5,201 cases and 9,066 controls
UC	28067908	de Lange KM et al	European	12,366 cases and 33,609 controls
T1D	32005708	Forgetta V et al	European	9,266 cases and 15,574 controls
PsO	23143594	Tsoi LC et al	European	10,588 cases and 22,806 controls
PSC	27992413	IPSCSG	European	2,871 cases and 12,019 controls
PBC	34033851	Cordell HJ et al	European	8,021 cases and 16,489 controls
AS	23749187	Cortes A et al	European	9,069 cases and 1,550 controls
ViT	27723757	Jin Y et al	European	2,853 cases and 37,405 controls
BE	36653562	FinnGen Consortium	European	2,188 cases and 311,286 controls
Adjustment of the model				
LDL-C	24097068	GLGC	96% European	173,082 individuals
HDL-C	24097068	GLGC	96% European	187,167 individuals
TG	24097068	GLGC	96% European	177,861 individuals
25OHD levels	32059762	Manousaki D et al.	European	441,291 individuals
Smoking	30643251	GSCAN	European	1,200,000 individuals
BMI	30239722	GIANT	European	694,649 individuals

BMI, body mass index; GWAS and Sequencing Consortium of Alcohol and Nicotine use; GIANT: Genetic Investigation of Anthropometric Traits; CD, Crohn's disease; CeD, Celiac disease; MS, Multiple sclerosis; RA, Rheumatoid arthritis; SLE, Systemic lupus erythematosus; UC, Ulcerative colitis; T1D, Type 1 diabetes; PsO, Psoriasis; PSC, Primary sclerosing cholangitis; PBC, Primary biliary cirrhosis; AS, Ankylosing spondylitis; ViT, Vitiligo; BE, Bronchiectasis; LDL-C, Low Density Lipoprotein Cholesterol; HDL-C, High Density Lipoprotein Cholesterol; TG, Triglyceride; 25OHD, 25-hydroxyvitamin D; GLGC, Global Lipids Genetics Consortium; IMSGC, International Multiple Sclerosis Genetics Consortium; IPSCSG, International PSC Study Group; Ref, reference (PUBMED ID).

It is imperative to note that all encompassed GWAS investigation procured endorsements from the relevant academic oversight committees. Given that our study was based on a secondary analysis of publicly disclosed datasets, further ethical vetting was not required.

In order to preserve the integrity of our Mendelian Randomization approximations, the chosen Single Nucleotide Polymorphisms (SNPs) were obligated to align with the ensuing benchmarks:

#### Genetic instrument selection

(1) Each of the SNPs selected as IVs established a notable resonance with stipulated exposure at a genome-wide significance threshold (*p*< 5×10^-8^).
(2) Rigorous scrutiny ensured that the SNPs did not have associations with possible confounders nor shared interdependence, thereby mitigating biases originating from linkage disequilibrium (r^2^ < 0.001, clumping distance = 10,000 kb).

#### Genetic instrument validation

(3) We used F-statistics (where F = beta²/se², with beta symbolizing the SNP-exposure nexus and variance denoted by se) to assess the potency of the instrumental variables (14). An elevated F-statistic indicates pronounced instrumental vigor. Consequently, it was essential that all integrated SNPs exhibit an F-statistic transcending 10.
(4) We used the MR-Steiger filtration method to enhance the reliability of our conclusions, thereby ruling out variables that are more related to the outcomes than exposures (15).
(5) In the event of an SNP’s absence from the outcome database, we used the SNiPa digital repository (accessible at http://snipa.helmholtz-muenchen.de/snipa3/) to locate a particular SNP. This platform used genotype data from a European cohort obtained from the 1000 Genomes Project Phase 3. Therefore, a surrogate SNP, reflecting linkage disequilibrium (r2 > 0.8) with the primary SNP was identified.
(6) The SNP’s footprint on exposure juxtaposed with its impact on the outcome must mirror the identical allele. An SNP found to be discordant in this regard was invariably excised.

### Source of exposure and outcome phenotypes

For autoimmune diseases, all from large abstract-level GWAS studies, ulcerative colitis (UC) and Crohn’s disease (CD) from de Lange KM et al. (16), celiac disease (CeD) from Trynka et al. (17), multiple sclerosis (MS) from International Multiple Sclerosis Genetics Consortium (IMSGC) (18), RA from Ha E et al. (19), systemic lupus erythematosus (SLE) from Bentham J et al. (20), type 1 diabetes (T1D) from Forgetta V et al. (21), psoriasis (PsO) from Tsoi LC et al. (22), primary sclerosing cholangitis (PSC) from International PSC Study Group (IPSCSG) (23), primary biliary cirrhosis (PBC) from Cordell HJ et al. (24), ankylosing spondylitis (AS) from Cortes A et al. (25), vitiligo (ViT) from Jin Y et al. (26), and for the outcome phenotype BE from FinnGen (R9) Consortium (27).

### Data sources for possible mediators

We further obtained genetic associations for Body Mass Index (BMI) from the Genetic Investigation of Anthropometric Traits (GIANT) consortium (28), smoking from GWAS and Sequencing Consortium of Alcohol and Nicotine use (GSCAN) (29), triglycerides (TG), Low Density Lipoprotein Cholesterol (LDL-C) and High Density Lipoprotein Cholesterol (HDL-C) from Global Lipids Genetics Consortium (GLGC) (30), 25-hydroxyvitamin D (25OHD) levels from Manousaki D et al. (31).

## Statistical analyses

### Primary MR analysis

For the UVMR study, the Wald ratio test was used for exposure with only one instrument, and the multiplicative random-effects inverse-variance-weight (IVW) method was implemented for the causative assessment of multiple IVs (comprising two or more). This approach was further enhanced by incorporating both the MR-Egger and weight median techniques. The weightage in IVW is directly related to each SNP’s Wald ratio estimate and inversely correlated with the variance estimate of each SNP’s Wald ratio (32). When all genetic markers are judged valid, IVW provides estimates that are both consistent and efficient. Conversely, the weight median method stands out when over half of the genetic markers are deemed questionable, and the MR-Egger approach is adopted when all genetic markers are refutable (33). Stringent adjustment for multiple comparisons was performed using the False Discovery Rate (FDR). Following this adjustment, a *P*-value < 0.05 was considered indicative of a significant causal relationship. However, instances where the raw *P*-value was below 0.05, but the FDR-adjusted P-value exceeded this threshold were regarded as tentative.

Given the potential confounding effects of factors, such as BMI, smoking habits, lipid profiles (LDL-C, HDL-C, and TG), and 25OHD levels on the progression from exposure to outcome, subsequent MVMR analyses were performed. This study aimed to accurately quantify the direct causative effects of exposure on the results. When juxtaposed with the UVMR paradigm, the primary supposition of MVMR focus on genetic variability associated with one or more exposures, whereas the succeeding assumptions harmonize with the UVMR framework (34). A refined investigation was undertaken to ascertain the magnitude of mediation by certain factors. The initial step was to obtain the MR effect projections for exposure in relation to the outcome phenotypes using the IVW approach. Thereafter, multivariate MR analysis was performed to ascertain the impact of nine mediating factors on the outcome while concurrently considering exposure attributes. The indirect influence of the exposure was determined by multiplying the resulting estimates for each outcome. Finally, the division of the mediation effect by the overarching effect provided insight into the relative contribution of the mediators to the overall outcome.

### Genetic correlation analysis

The LDSC regression, specifically tailored for GWAS summary data, serves as a robust approach for dissecting genetic correlations across complex diseases and traits. Notably, LDSC efficiently differentiates genuine polygenic signals from potential confounders such as cryptic relatedness and population stratification (35). A consequential genetic correlation, both statistically and quantitatively robust, signifies that an overarching phenotypic correlation is not merely attributable to environmental confounders (35). The LDSC tool, accessible at (https://github.com/bulik/ldsc), was used to scrutinize the genetic intersections between exposure and an array of outcome phenotypes.

### Sensitivity analysis

Within the framework of UVMR analysis, several tests were conducted to validate its rigor and authenticity. The heterogeneity of the selected genetic variants was assessed using Cochran’s Q test, wherein a *P*-value of < 0.05 indicated pronounced discrepancies among the scrutinized SNPs (36). Employing the MR-Egger regression (37), this investigation discerned the potential for directional pleiotropy within the MR context. MR-Egger’s intercept, with a *P*-value < 0.05, signified the presence of consequential directional pleiotropy despite the inherent limitations of this methodology (38). The MR Pleiotropy Residual Sum and Outlier (MR-PRESSO) approach was used to identify probable outliers and delve into horizontal pleiotropy, which was inferred when the global p-value was less than 0.05 (39). By excluding such outliers, the data correction was refined. An ensuing leave-one-out analysis elucidated the impact of singular SNPs on collective outcomes (40).

R^2^ was calculated using the formula 2×MAF×(1-MAF) ×beta^2^, where MAF denotes the minor allele frequency for each designated SNP. The cumulative values provided a coefficient essential for power computation (41). The determination of statistical potency was anchored on the mRnd platform (42) and is accessible at https://shiny.cnsgenomics.com/mRnd/.

## Results

### Genetic instrument selection and genetic correlation between phenotypes

The SNPs of each autoimmune disease were screened according to the genetic instrument selection process described above. Power calculations for bidirectional univariable MR analyses between autoimmune diseases including CD, CeD, MS, RA, SLE, UC, T1D, PsO, PSC, PBC, AS, ViT and BE, were performed. The study reported F- statistics exceeding 60 for all instrumental variants, signifying a robust reduction in bias from weak instruments. The SNPs selected as IVs ranged from 15 to 83, accounting for an explained variance of 2.59% to 1535.64% (**Supplementary Table 1**).

**Figure 2:**
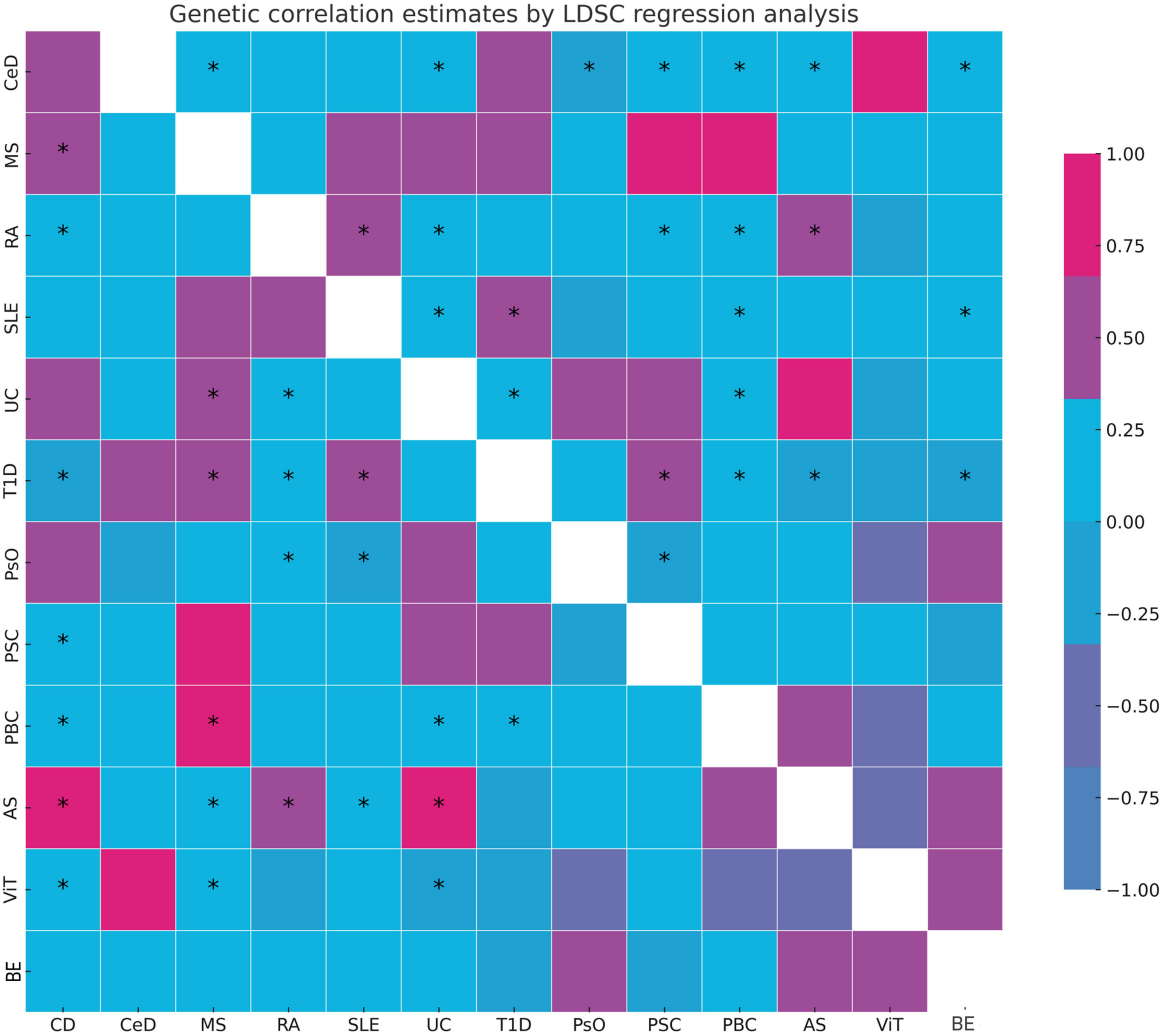
Summary of genetic correlation results. *: represents the presence of genetic correlation, *P*<0.05. LDSC, linkage disequilibrium score; CD, Crohn's disease; CeD, Celiac disease; MS, Multiple sclerosis; RA, Rheumatoid arthritis; SLE, Systemic lupus erythematosus; UC, Ulcerative colitis; TID, Type 1 diabetes; PsO, Psoriasis; PSC, Primary sclerosing cholangitis; PBC, Primary biliary cirrhosis; AS, Ankylosing spondylitis; ViT, Vitiligo; BE, Bronchiectasis.

LDSC genetic correlation analyses were conducted to estimate the genetic correlation between different autoimmune diseases and BE. LDSC analysis revealed significant genetic correlations between BE and CD (r_g_ = 0.220, *P* = 0.037), RA (r_g_ = 0.210, *P* = 0.021), and UC (r_g_ = 0.247, *P* = 0.023) (**Supplementary Table 2**). However, no genetic correlation was found with other autoimmune diseases (*P* > 0.05). The SNP-based liability-scale heritability (h²) ranged from 0.1% to 232.99%. Additionally, the genetic correlation between each autoimmune disease and BE was analyzed (**Figure 2**; **Supplementary Table 3**).

### Association of genetically predicted autoimmune diseases with BE

**Figure 3:**
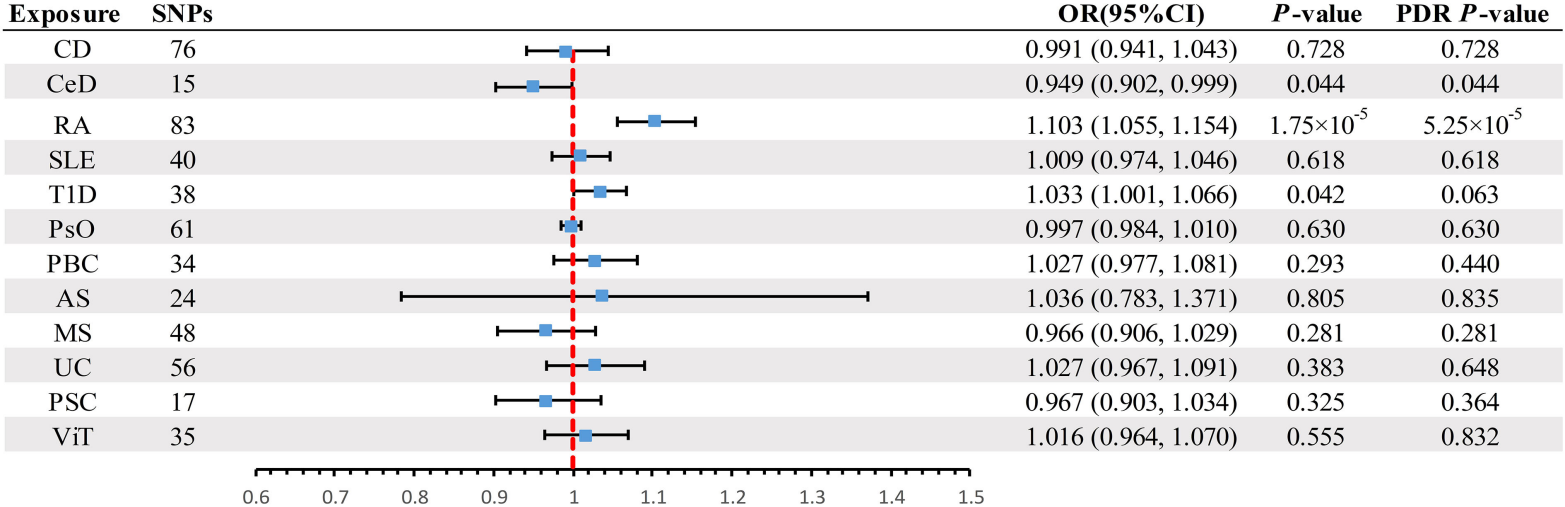
Summary of IVW results for the main UVMR analysis methods. IVW, Inverse variance weight; UVMR, Univariate Mendelian Randomization; SNP, Single Nucleotide Polymorphism; FDR, False Discovery Rate; OR, odds ratio; CI, confidence interval; CD, Crohn's disease; CeD, Celiac disease; MS, Multiple sclerosis; RA, Rheumatoid arthritis; SLE, Systemic lupus erythematosus; UC, Ulcerative colitis; T1D, Type 1 diabetes; PsO, Psoriasis; PSC, Primary sclerosing cholangitis; PBC, Primary biliary cirrhosis; AS, Ankylosing spondylitis; ViT, Vitiligo.

**Figure 4:**
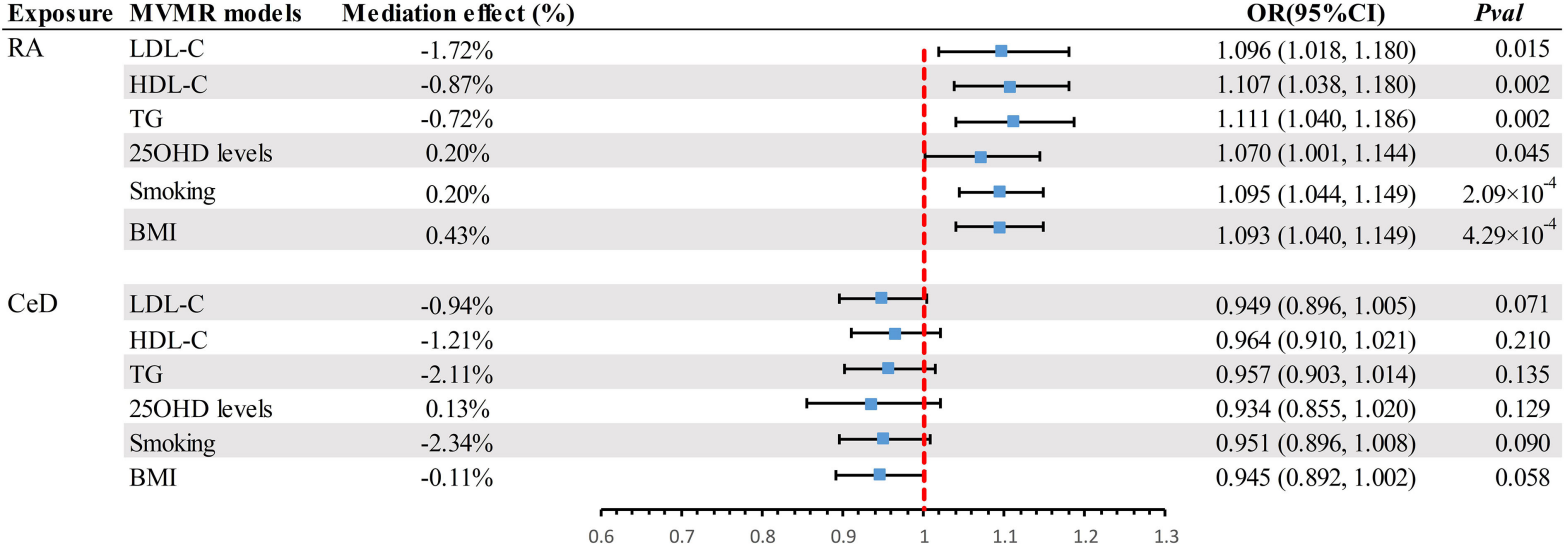
Summary of MVMR results. Estimating the impact of all positive exposure factors on the outcome through potential mediators. MVMR, Multivariate Mendelian Randomization; BMI, body mass index; OR, odds ratio; CI, confidence interval; LDL-C, Low Density Lipoprotein Cholesterol; HDL-C, High Density Lipoprotein Cholesterol; TG, Triglyceride; 250HD, 25-hydroxyvitamin D; CeD, Celiac disease; RA, Rheumatoid arthritis.

A scatter plot illustrates the causal relationship between each autoimmune disease and BE (**Supplementary Figure 1**). After adjusting for multiple comparisons, the primary IVW analysis provided strong evidence for two causal relationships (**Figure 3**). Specifically, for each standard deviation (SD) increase in genetically predicted RA, there was a 10.3% increase in the incidence of BE (odds ration [OR] = 1.103, 95% CI 1.055-1.154, *P* = 1.75×10^-5^, FDR = 5.25×10^-5^). Furthermore, for every SD increase in CeD, the incidence of BE was reduced by 5.1% (OR = 0.949, 95% CI 0.902-0.999, *P* = 0.044, FDR = 0.044). We also observed suggestive evidence corresponding to a 3% increase in BE incidence with T1DM (OR = 1.033, 95% CI 1.001-1.066, *P* = 0.042, FDR = 0.063). Additionally, we had 96%, 100%, and 92% statistical power to detect the associations of CeD, RA, and T1D with BE, with OR values of 1.103, 0.949, and 1.033, respectively (**Supplementary Table 1**). No other causal relationship evidence was found (P > 0.05, FDR > 0.05) (**Table 2**). Furthermore, MVMR analysis showed that RA was an independent risk factor for BE, whereas mediator MR analysis did not identify any mediating factors (**Figure 4**).

**Table 2:** Summary of UVMR analysis results.

Methods	SNPs	Crohn's disease (CD)						SNPs	Celiac disease (CeD)					
		OR	or_lci95	or_uci95	beta	P-value	FDR		OR	or_lci95	or_uci95	beta	P-value	FDR
Inverse variance weight	76	0.991	0.941	1.043	-0.009	0.728	0.728	15	0.949	0.902	0.999	-0.052	0.044	0.044
MR Egger	76	0.939	0.819	1.078	-0.063	0.375	0.5625	15	0.917	0.852	0.987	-0.087	0.039	0.044
Weight median	76	0.956	0.886	1.031	-0.045	0.238	0.5625	15	0.933	0.882	0.987	-0.069	0.015	0.044

Methods	SNPs	Rheumatoid arthritis (RA)						SNPs	Systemic lupus erythematosus (SLE)					
		OR	or_lci95	or_uci95	beta	P-value	FDR		OR	or_lci95	or_uci95	beta	P-value	FDR
Inverse variance weight	83	1.103	1.055	1.154	0.098	1.75E-05	5.25E-05	40	1.009	0.974	1.046	0.009	0.618	0.618
MR Egger	83	1.098	1.026	1.175	0.094	0.008	1.20E-02	40	0.954	0.884	1.031	-0.047	0.242	0.5055
Weight median	83	1.087	1.012	1.167	0.083	0.022	2.20E-02	40	1.027	0.973	1.084	0.027	0.337	0.5055

Methods	SNPs	Type 1 diabetes (T1D)						SNPs	Psoriasis (PsO)					
		OR	or_lci95	or_uci95	beta	P-value	FDR		OR	or_lci95	or_uci95	beta	P-value	FDR
Inverse variance weight	38	1.033	1.001	1.066	0.033	0.042	0.063	61	0.997	0.984	1.010	-0.003	0.630	0.63
MR Egger	38	1.071	1.021	1.122	0.068	0.007	0.021	61	0.991	0.975	1.008	-0.009	0.312	0.468
Weight median	38	1.035	0.990	1.082	0.034	0.131	0.131	61	0.989	0.973	1.005	-0.011	0.182	0.468

Methods	SNPs	Primary biliary cirrhosis (PBC)						SNPs	Ankylosing spondylitis (AS)					
		OR	or_lci95	or_uci95	beta	P-value	FDR		OR	or_lci95	or_uci95	beta	P-value	FDR
Inverse variance weight	34	1.027	0.977	1.081	0.027	0.293	0.4395	24	1.036	0.783	1.371	0.035	0.805	0.835
MR Egger	34	1.051	0.915	1.208	0.050	0.484	0.484	24	1.259	0.783	2.026	0.231	0.352	0.835
Weight median	34	1.080	1.004	1.163	0.077	0.040	0.12	24	1.041	0.715	1.515	0.040	0.835	0.835

Methods	SNPs	Multiple sclerosis (MS)						SNPs	Ulcerative colitis (UC)					
		OR	or_lci95	or_uci95	beta	P-value	FDR		OR	or_lci95	or_uci95	beta	P-value	FDR
Inverse variance weight	48	0.966	0.906	1.029	-0.035	0.281	0.281	56	1.027	0.967	1.091	0.027	0.383	0.648
MR Egger	48	0.886	0.780	1.007	-0.121	0.070	0.105	56	1.043	0.872	1.246	0.042	0.648	0.648
Weight median	48	0.912	0.829	1.004	-0.092	0.059	0.105	56	1.030	0.942	1.126	0.029	0.516	0.648

Methods	SNPs	Primary sclerosing cholangitis (PSC)						SNPs	Vitiligo (ViT)					
		OR	or_lci95	or_uci95	beta	P-value	FDR		OR	or_lci95	or_uci95	beta	P-value	FDR
Inverse variance weight	17	0.967	0.903	1.034	-0.034	0.325	0.364	35	1.016	0.964	1.070	0.016	0.555	0.8325
MR Egger	17	0.883	0.792	0.984	-0.124	0.040	0.12	35	1.140	0.949	1.369	0.131	0.170	0.51
Weight median	17	0.963	0.887	1.045	-0.038	0.364	0.364	35	0.995	0.926	1.070	-0.005	0.903	0.903

To avoid excessive bias effects, Cochran’s Q test was performed to analyze the sensitivity of the MR results, and no evidence of heterogeneity was observed (*P>0.05*). Moreover, no horizontal pleiotropy was identified using the MR-Egger intercept test (*P>0.05*) or the MR-PRESSO global test (*P>0.05*). These analyses confirmed the robustness of the findings (**Table 3**). Leave-one-out analysis did not reveal any horizontal pleiotropy and further confirmed that the causal relationship was not influenced by any individual SNP (**Supplementary File 1**).

**Table 3:** Summary of sensitivity results.

Exposure	Outcome	MR-Egger intercept			MR-PRESSO global test			Cochrane’s Q			Steiger_test		
		Intercept	SE	*Pval*	RSS_obs_	*P*-value	*Outlier*	*Q*	*Q_df*	*Q_pval*	Direction	*Pval*	Filtered SNPs
CD	BE	0.009	0.011	0.414	84.328	0.300	*NA*	81.699	75	0.279	TRUE	0	NA
CeD		0.020	0.016	0.238	20.231	0.257	*NA*	17.773	14	0.217	TRUE	0	NA
MS		0.016	0.011	0.136	45.697	0.630	*NA*	42.816	47	0.646	TRUE	0	NA
RA		0.001	0.006	0.858	84.445	0.484	*NA*	82.900	82	0.451	TRUE	0	NA
SLE		0.022	0.014	0.118	37.818	0.551	*NA*	35.118	39	0.648	TRUE	0	NA
UC		-0.001	0.014	0.966	48.172	0.657	*NA*	46.698	55	0.780	TRUE	0	NA
T1D		-0.020	0.010	0.054	31.404	0.811	*NA*	29.506	37	0.805	TRUE	0	NA
PsO		0.012	0.012	0.309	80.749	0.061	*rs73695700*	78.790	60	0.052	TRUE	0	NA
PSC		0.040	0.020	0.064	27.022	0.113	*NA*	22.246	16	0.135	TRUE	0	NA
PBC		-0.006	0.018	0.729	42.556	0.190	*NA*	38.110	33	0.248	TRUE	0	NA
AS		-0.013	0.013	0.330	28.893	0.331	*NA*	26.232	23	0.290	TRUE	0	NA
ViT		-0.031	0.024	0.207	41.969	0.251	*rs28688825*	39.443	34	0.240	TRUE	0	NA

All results are after removing outliers and re-running the MR analysis. CD, Crohn's disease; CeD, Celiac disease; MS, Multiple sclerosis; RA, Rheumatoid arthritis; SLE, Systemic lupus erythematosus; UC, Ulcerative colitis; T1D, Type 1 diabetes; PsO, Psoriasis; PSC, Primary sclerosing cholangitis; PBC, Primary biliary cirrhosis; AS, Ankylosing spondylitis; ViT, Vitiligo; BE, Bronchiectasis; SNP, Single Nucleotide Polymorphisms.

## Discussion

In this study, we performed a comprehensive MR analysis to investigate the relationship between autoimmune diseases and BE. The results of LDSC analysis revealed significant genetic correlations between BE and CD, RA, and UC. However, beyond the aforementioned genetic correlations, no other genetic correlations were observed. Moreover, our objective in utilizing the MR analysis was to mitigate bias and confounding factors and identify causal associations. Interestingly, we found suggestive evidence of an association between T1D and BE. The MVMR analysis substantiated RA as an independent risk factor for BE, whereas the mediation MR analysis did not reveal any mediating model. While observational studies have inherent limitations, such as potential confounders and ambiguous causality, our MR approach aimed to mitigate these biases, providing clarity to these associations.

BE is characterized by damaged and dilated bronchi and is one of the most common pulmonary manifestations in patients with RA (43). Persistent pulmonary inflammation can inflict irreversible damage to the bronchi, culminating in BE (44). This notion is further supported by Lake et al., who suggested that pulmonary nodules, pleurisy, and air trapping in patients with RA might elevate the risk of anomalous pulmonary dilation (45). Additionally, Jin et al. found that the systemic inflammatory milieu in patients with RA might increase their susceptibility to other inflammatory disorders (46). Such inflammation can impair the bronchial walls, leading to BE. Moreover, Quirke et al. demonstrated that BE is a potent model for the initiation of autoimmunity in RA via bacterial infection of the lungs (47). CeD pathophysiologically correlates with autoimmune damage to the small intestine (48). This autoimmune response can potentially affect the lungs, wherein damage to the intestine may precipitate the migration of inflammatory cells to the lungs, causing bronchitis. Dellaripa et al. also drew attention to dysregulated immune responses, suggesting that lungs are potential targets for autoimmune diseases (49). The primary hallmark of T1D is hyperglycemia, which stems from an immune attack on pancreatic β-cells. Barrett et al. suggested that microvascular damage correlated with T1DM might compromise the airway blood supply, contributing to BE (50). Lewis et al. have found that cystic fibrosis-associated diabetes (CFRD) often leads to poorer clinical outcomes in patients with CF including increased in pulmonary exacerbations, poorer lung function, and early mortality (51).

Emerging research has probed possible shared genetic pathways between autoimmune diseases and BE. Juge et al. have identified shared genetic susceptibilities between RA and respiratory ailments (52). Moreover, both CeD and T1D have been linked to gut microbiota dysbiosis (53, 54). An MR study by Huang et al. delineated a causal relationship between the gut microbiome and pulmonary diseases (55), hinting at the potential influence of the gut microbiota on pulmonary health and the predisposition to BE. Finally, as discussed by Litman et al., certain medications for autoimmune diseases may inadvertently exacerbate or induce pulmonary conditions (56).

The differences in the results between the MR and LDSC may be attributed to their distinct methodologies. MR relies on the use of genetic variants as instruments to infer causality, which assumes that these genetic variants affect the outcome solely through their impact on the exposure of interest and are not influenced by unmeasured confounding factors. Differently, LDSC focuses on quantifying genetic similarities between phenotypes and diseases. A significant genetic correlation detected by LDSC indicated shared genetic variations across multiple loci between the phenotypes. However, it is important to note that LDSC does not necessarily imply a causal relationship. In light of our findings, it is evident that there may be a causal relationship between BE and RA, and direct genetic correlations were detected using LDSC.

Our study has several strengths. First, our MR approach holistically analyzed the causative relationships between autoimmune diseases and BE. Second, the unique identification of SNPs as IVs in the European population minimized potential population stratification biases. Third, we employed rigorous methods with an F-statistic exceeding 10, reducing the biases from weak instruments. Fourth, we evaluated the confounding influence of the MVMR. Fifth, we relied on myriad sensitivity analyses based on statistical models and ‘leave-one-out’ techniques to enhance the reliability of the results. However, this study has several limitations. First, because of the lack of IVs achieving genome-wide significance for the outcomes, reverse causation inference was unfeasible. Second, summary-level GWAS data precluded subgroup analyses of autoimmune diseases and BE. Third, the sequencing and analysis methods for each autoimmune disease and BE may differ, contributing to the distinct results. Lastly, due to the summary-level GWAS data, the demographic data of the studies are absent, and further subgroup analysis of confounding factors, such as age and gender on autoimmune diseases and BE remains unknown.

## Conclusion

LDSC analysis suggested significant genetic correlations between several autoimmune diseases and BE, and further MVMR analysis showed that RA was an independent risk factor for BE. These results provide genetic evidence for further mechanistic and clinical studies aimed at understanding the association between BE and autoimmune diseases.

## Data availability statement

The original contributions presented in the study are included in the article/**Supplementary Material**. Further inquiries can be directed to the corresponding author.

## Author contributions

YS: Conceptualization, Data curation, Formal analysis, Methodology, Writing – original draft. YZ: Conceptualization, Data curation, Formal analysis, Methodology, Software, Writing – original draft. YC: Writing – original draft. JX: Conceptualization, Resources, Supervision, Writing – review & editing.
