# Supplementary material for: Autoimmune diseases and their genetic link to bronchiectasis: insights from a genetic correlation and Mendelian randomization study
Source: Front Immunol. 2024 Apr 10;15:1343480. doi: 10.3389/fimmu.2024.1343480 (PMC11039849; doi:10.3389/fimmu.2024.1343480)
Supplement: Supplementary file 1 [file DataSheet_1.pdf]

Table of Contents

----

1. Description of Summary Statistics Data Sources 1-11

2. Fig. 1 Summary of 12 Autoimmune Disease Funnel Charts 12

3. Fig. 2-13 Summary of 12 Autoimmune Disease Leave-One-Out Method Analyses.......13-24

4. Fig. 14-25 Summary of Forest Graph Analysis of 12 Autoimmune Diseases.......25-36

5. Reference 37-38

Description of summary statistics data sources

GWAS of ulcerative colitis (UC) and Crohn's disease (CD) by de Lange KM et al[1].

Ulcerative colitis (UC) and Crohn's disease (CD) are two chronic inflammatory bowel diseases that affect millions of people worldwide. In recent years, there has been a significant increase in the number of genome-wide association studies (GWAS) aimed at identifying genetic risk factors for UC and CD. These studies have identified numerous genetic loci associated with UC and CD, but the underlying mechanisms of these associations are not yet fully understood.

To better understand the genetic basis of UC and CD, a team of researchers conducted a large-scale GWAS using data from multiple sources. The summary statistics data used in this study were obtained from a variety of sources, including previously published GWAS studies and newly generated data from the UK IBD Genetics Consortium and UK10K Consortium. The researchers used a variety of quality control measures to ensure that the data were of high quality and suitable for analysis. Samples with missing data, heterozygosity outside of the expected range, and other quality control issues were removed from the analysis. After quality control, the data were available for 4,474 CD cases, 4,173 UC cases, 592 IBD-unclassified cases, and 9,500 controls for 296,203 variants. The researchers then performed a meta-analysis of the new GWAS data with previously published summary statistics from 12,882 IBD cases and 21,770 population controls. This analysis identified 25 new loci at genome-wide significance for UC and CD, as well as several previously discovered loci that were genome-wide significant in the new data.

To identify causal variants, genes, and mechanisms, the researchers performed a summary-statistic fine-mapping analysis on these loci. This analysis revealed several potential causal variants and genes, as well as potential mechanisms underlying the associations. One of the most significant findings of the study was a genome-wide significant association on chromosome 10q25 that was previously only associated with CD in individuals of East Asian ancestry. This finding supports the idea that there is near complete sharing of genetic risk loci across populations. The researchers also observed inflation of the summary statistics, but LD score regression demonstrated that this was due to broad polygenic signal, rather than confounding population substructure. This suggests that the associations identified in the study are likely to be genuine and not the result of population stratification.

Overall, this study provides important new insights into the genetic basis of UC and CD and highlights the need for further research to fully understand the underlying mechanisms of these associations. The summary statistics data used in this

GWAS of celiac disease (CeD) by Trynka et al[2]

The summary statistics data sources used in this GWAS were obtained from a large-scale genotyping project that included over 40,000 individuals of European ancestry. The genotyping was performed using the Illumina Immunochip, which is a custom genotyping array that includes over 200,000 single nucleotide polymorphisms (SNPs) that have been associated with autoimmune diseases.

The GWAS included a total of 9,407 cases of CeD and 17,791 controls. The cases were diagnosed based on a combination of clinical symptoms, serological tests, and biopsy results. The controls were individuals who did not have a history of CeD or any other autoimmune disease. The analysis of the GWAS data identified 13 loci that were associated with CeD at genome-wide significance (P<5×10-8). Of these loci, 10 were previously reported in other GWAS studies, while 3 were novel. The novel loci were located on chromosomes 3, 12, and 18.

The most significant association was observed at the HLA-DQA1 locus on chromosome 6, which has been previously identified as a major risk factor for CeD. In addition to the HLA region, the analysis also identified several non-HLA loci that were associated with CeD, including the IL18RAP, TAGAP, and PLEK loci.

The analysis also revealed that the genetic architecture of CeD is complex, with both common and rare variants contributing to disease risk. The rare variants were identified through a separate analysis of exome sequencing data from a subset of the CeD cases and controls.

GWAS of multiple sclerosis (MS) by International Multiple Sclerosis Genetics Consortium (IMSGC)[3]

In recent years, genome-wide association studies (GWAS) have been conducted to identify genetic variants associated with MS susceptibility. These studies have identified more than 50 non-MHC susceptibility alleles, but many additional susceptibility alleles remain to be identified.

One such study involved the analysis of immune-related loci using the ImmunoChip custom genotyping array. This study involved a large number of subjects and controls and identified 48 new susceptibility variants for MS. The initial analysis identified 13 secondary and 2 tertiary statistically independent signals using forward stepwise logistic regression. A total of 150 statistically independent association signals were identified, with 48 of these reaching a genome-wide significance p-value $<5 \times 10^{-8}$ at the discovery phase alone.

The findings of this study were validated through replication in 14,802 MS subjects and 26,703 healthy controls with available GWAS data imputed to the 1000 Genomes European phase I panel. A joint analysis of the discovery and replication phases was then performed to identify the 48 new susceptibility variants. In addition to identifying new susceptibility variants, this study also compared the top variants reported as either novel or previously known in other ImmunoChip reports with the 110 variants representing both the novel and previous discoveries in MS. An r2 ≥ 0.8 using the Pairwise LD function of the SNAP tool in European samples was required for a signal to be considered as overlapping. Secondary analyses were also performed, including a severity-based analysis of MSSS in cases only from the discovery phase and a transmission disequilibrium test in 633 trios to test for transmission of the 97 identified risk alleles.

The identification of these new susceptibility variants has significant implications for the understanding and treatment of MS. These variants may provide new targets for drug development and personalized treatment approaches. Additionally, the identification of these variants may lead

to a better understanding of the underlying mechanisms of MS and may help to identify new pathways for therapeutic intervention.

GWAS of rheumatoid arthritis (RA) by Ha E et al[4]

This particular study aimed to perform a large-scale meta-analysis of RA GWAS summary statistics data from East Asian and European populations. The data sources used in this study were three large case-control collections consisting of 311,292 individuals of Korean, Japanese, and European populations. The sample size of each GWAS was considerable, with 4068 RA cases and 36,487 controls in the Korean population, 4199 cases and 208,254 controls in the Japanese population, and 14,361 cases and 43,923 controls in the European population. The final sample size of the meta-analysis was more than 310,000.

The association summary statistics were retrieved from previous GWASs in Korean, Japanese, and European populations. The summary statistics included information on the effect size, standard error, and p-value of each genetic variant. The summary statistics were then used in an inverse-variance-weighted fixed-effects meta-analysis to identify new RA susceptibility loci and to better understand the genetic architecture of RA.

The results of the meta-analysis identified 11 new RA susceptibility loci that explained 6.9% and 1.8% of the single-nucleotide polymorphism-based heritability in East Asians and Europeans, respectively. In addition, the study confirmed 71 known non-human leukocyte antigens (HLA) susceptibility loci, identifying 90 independent association signals. The integration of accumulated knowledge of RA variants with emerging high-throughput omics data facilitated various post-GWAS approaches that helped unravel the biology of RA based on disease-risk variants in actual human patients, suggesting potentially repurposable drugs for RA treatment.

To narrow down the potentially functional variants, the study found 130 proxy variants in high LD ($r2 \geq 0.9$) with lead variants in both East Asian and European populations or in East Asians. These proxy variants were used to prioritize causal variants and genes, RA variant-implicating features (tissues, pathways, and transcription factors), and potentially repurposable drugs for RA treatment.

GWAS of systemic lupus erythematosus (SLE) by Bentham J et al[5]

The GWAS of SLE comprised 7,219 cases and 15,991 controls of European ancestry. The study consisted of a new GWAS, a meta-analysis with a published GWAS, and a replication study. The authors mapped 43 susceptibility loci, including 10 novel associations. Imputation provided evidence for missense variants underpinning associations in eight genes. Other likely causal genes were established by examining associated alleles for cis-acting eQTL effects in a range of ex vivo immune cells.

The authors found an over-representation of transcription factors among SLE susceptibility genes, supporting the view that aberrantly regulated gene expression networks in multiple cell types in both the innate and adaptive immune response contribute to the risk of developing SLE. The study also identified several novel pathways involved in the pathogenesis of SLE, including the JAK-STAT signaling pathway, the TGF-beta signaling pathway, and the B cell receptor signaling

pathway.

The data sources used in the GWAS of SLE included several publicly available databases and resources. The TwinsUK samples were obtained from the Department of Twin Research at King's College London. The Ingenuity Pathway Analysis was used to identify novel pathways involved in the pathogenesis of SLE. Immunobase was used to deposit summary statistics from the GWAS. The data will be made available to the scientific community for further analysis and interpretation.

In addition to the publicly available data sources, the authors also used their own in-house resources to analyze the data. The Systems Biology and Complex Disease Genetics group at King's College London provided expertise in the analysis of complex genetic data. The authors also used their own software tools to perform quality control, imputation, and association testing. The authors declare no competing financial interests in the study. The Europe PMC Funders Group provided financial support for the study. The manuscript was published in the journal Nature Genetics and is available for further reading and analysis.

GWAS of type 1 diabetes (T1D) by Forgetta V et al[6]

Type 1 diabetes (T1D) is a chronic autoimmune disease that affects millions of people worldwide. It is characterized by the destruction of insulin-producing beta cells in the pancreas, leading to a deficiency of insulin and high blood sugar levels. In recent years, there has been a growing interest in understanding the genetic basis of T1D, with the hope of developing new treatments and preventative measures for this debilitating disease.

One of the most promising approaches to studying the genetics of T1D is through genome-wide association studies (GWAS). These studies involve analyzing the DNA of large numbers of individuals with and without T1D to identify genetic variants that are associated with the disease. By comparing the genomes of these two groups, researchers can identify regions of the genome that are more common in people with T1D, and use this information to develop new insights into the underlying biology of the disease. The summary statistics data sources used in this GWAS were drawn from 12 cohorts of predominantly European descent, totaling 9,358 T1D case subjects and 15,705 control subjects. The individual-level genotype data for the discovery meta-analysis were drawn from a variety of sources, including the Genetics of Kidneys in Diabetes (GoKinD) study, the Wellcome Trust Case Control Consortium (WTCCC), and the Type 1 Diabetes Genetics Consortium (T1DGC).

In the discovery stage of the GWAS, the researchers used 9,684 T1D case subjects and 17,153 control subjects to identify genetic variants that were associated with the disease. Specifically, they analyzed over 9 million single nucleotide polymorphisms (SNPs) to identify regions of the genome that were more common in people with T1D. They then replicated their findings using de novo genotyping in a separate cohort of 4,329 T1D case subjects from the T1DGC and 9,543 control subjects from the UK Biobank.

The researchers identified a total of 42 independent loci that were associated with T1D, including three that were previously unknown. They also found that the effect sizes of the rare variants they

identified were larger than those of common variants in other loci. This suggests that rare variants may play a more important role in the development of T1D than previously thought.

GWAS of psoriasis (PsO) by Tsoi LC et al[7].

Psoriasis is a chronic autoimmune disease that affects the skin, nails, and joints. It is characterized by red, scaly patches on the skin that can be itchy and painful. The exact cause of psoriasis is unknown, but it is believed to be a combination of genetic and environmental factors. In recent years, there has been a growing interest in understanding the genetic basis of psoriasis, with the hope of developing more effective treatments.

To this end, a meta-analysis of three genome-wide association studies (GWAS) and two independent datasets genotyped on the Immunochip was conducted. The study involved a total of 10,588 cases and 22,806 controls of European Caucasian descent. The aim of the study was to gain further insight into the genetic architecture of psoriasis and to identify new disease susceptibility regions.

The GWAS data sets used in the study were the Kiel, CASP, and WTCCC2 data sets, which were previously described. The samples of the Psoriasis/Arthritis Genetics Extension (PAGE) and the Genetic Analysis of Psoriasis Consortium (GAPC) datasets were collected from subjects of European Caucasian descent at the participating institutions after obtaining informed consent in adherence with the Declaration of Helsinki Principles. DNA was isolated from blood or EBV-immortalized lymphoblastoid cell lines using standard methods.

For each GWAS, the SNP density was increased through imputation by using European haplotype sequences generated by the 1000 Genomes Project as templates. Overall, the analysis included 111,236 SNPs that were genotyped in both Immunochip datasets and also had good imputation quality in at least two of the three GWAS.

The meta-analysis of all five datasets yielded genome-wide significance for 19 of the 21 known psoriasis loci. The remaining two loci, ZMIZ1 and PRDX5, showed nominal evidence in the combined analysis. In addition, 15 new risk loci were identified at $P < 5×10-8$. The newly identified shared disease regions encompassed a number of genes whose products regulate T-cell function, such as RUNX3, TAGAP, and STAT3. The new psoriasis-specific regions were notable for candidate genes whose products are involved in innate host defense, encoding proteins with roles in interferon-mediated antiviral responses (DDX58), macrophage activation (ZC3H12C), and NF-κB signaling (CARD14 and CARM1).

GWAS of primary sclerosing cholangitis (PSC) from International PSC Study Group (IPSCSG)[8].

Primary sclerosing cholangitis (PSC) is a rare and progressive disorder that leads to bile duct destruction. It is a chronic liver disease that affects both men and women, and it is often associated with inflammatory bowel disease (IBD). PSC is characterized by inflammation and fibrosis of the bile ducts, which can lead to cirrhosis, liver failure, and the need for liver transplantation.

To better understand the genetic basis of PSC, a large-scale genome-wide association study (GWAS) was conducted. The GWAS included 4,796 cases of PSC and 19,955 population controls, making it the largest study of its kind to date. The study identified four novel genome-wide significant loci associated with PSC, as well as several previously identified loci.

The summary statistics data sources used in this GWAS were obtained from a variety of public databases and research studies. These sources included the 1000 Genomes Project, the UK10K Project, and several other GWAS studies of PSC and related diseases. The 1000 Genomes Project is a global reference for human genetic variation, and it provided the researchers with a comprehensive catalog of genetic variants across different populations. The UK10K Project, on the other hand, focused on identifying rare genetic variants in health and disease. By combining data from these two projects, the researchers were able to identify both common and rare genetic variants associated with PSC.

In addition to these large-scale projects, the researchers also used data from several smaller GWAS studies of PSC and related diseases. These studies provided valuable insights into the genetic basis of PSC and helped to validate the findings of the larger GWAS. To analyze the data from these various sources, the researchers used a variety of statistical methods and software tools. One such tool was METAL, which is a fast and efficient program for meta-analysis of genome-wide association scans. METAL allowed the researchers to combine data from multiple studies and identify genetic variants that were consistently associated with PSC across different populations.

Overall, the summary statistics data sources used in this GWAS provided a comprehensive and diverse set of genetic variants for analysis. By combining data from multiple sources and using advanced statistical methods, the researchers were able to identify several novel genetic loci associated with PSC. These findings have important implications for understanding the genetic basis of PSC and developing new treatments for this rare and debilitating disease.

GWAS of primary biliary cirrhosis (PBC) by Cordell HJ et al[9]

This genome-wide meta-analysis study on primary biliary cholangitis (PBC) utilized data from a total of seven cohorts, including five cohorts of European ancestry and two East Asian cohorts. The study included a total of 10,516 cases and 20,772 controls, making it one of the largest GWAS studies on PBC to date.

The data sources for this study were carefully selected to ensure a diverse and representative sample of PBC cases and controls. The European cohorts included the UK-PBC Research Cohort, the Italian PBC Study Group, the French PBC Study Group, the German PBC Study Group, and the Swedish PBC Study Group. The East Asian cohorts included the Japanese PBC Study Group and the Chinese PBC Study Group. The UK-PBC Research Cohort was genotyped at the Wellcome Sanger Institute, while the other cohorts were genotyped using Illumina arrays. The genotyping data was then subjected to quality control measures to ensure accuracy and reliability.

In addition to the genotyping data, this study also utilized ‘omic data, including methylome-wide

and transcriptome-wide data, to guide the selection of candidate genes for PBC. This integration of GWMA statistics with ‘omic data allowed for a more comprehensive analysis of the genetic architecture of PBC and the identification of potential drug targets. The summary statistics data from this study will be deposited with the European Genome-phenome Archive following publication. This will allow other researchers to access and utilize the data for further analysis and validation.

Overall, this study provides valuable insights into the genetic architecture of PBC and identifies potential drug targets for the treatment of this chronic liver disease. The careful selection of diverse and representative cohorts, as well as the integration of ‘omic data, ensures the reliability and accuracy of the summary statistics data.

GWAS of ankylosing spondylitis (AS) by Cortes A et al[10].

Ankylosing spondylitis (AS) is a chronic inflammatory disease that primarily affects the spine and sacroiliac joints. It is a complex disease with both genetic and environmental factors contributing to its development. In recent years, genome-wide association studies (GWAS) have been used to identify genetic variants associated with AS. In this 2000-word summary, we will provide a detailed description of the summary statistics data sources used in a recent GWAS of AS.

The GWAS of AS was conducted by an international consortium of researchers, who analyzed data from a total of 12,252 DNA samples. All cases had definite AS according to the modified New York criteria, and written informed consent was obtained from all cases with approval from the relevant research ethics authorities at each participating center. The case collection consisted of 10,417 individuals of European ancestry, 1,560 of Asian ancestry, and 275 from Latin America (Colombia and Mexico). Of these, 2,425 cases of European ancestry have previously been reported in GWAS. The researchers obtained 12,338 controls of European ancestry, 1,570 of East Asian ancestry, and 445 from Latin America. These included shared controls from the UK 1958 Birth Cohort, the UK Blood Services Common Controls, and the United States and from participating centers from France, The Netherlands, Norway, Spain, Mexico, Colombia, China, Taiwan, and Korea.

The genotyping was performed using the Illumina Immunochip microarray, which contains over 196,000 single nucleotide polymorphisms (SNPs) and insertion/deletion polymorphisms (indels) that are enriched for immune-related loci. Quality control was performed on the genotyping data, and samples with a call rate of less than 95% were excluded. SNPs with a call rate of less than 95%, a minor allele frequency of less than 1%, or a Hardy-Weinberg equilibrium p-value of less than 1×10^{-6} were also excluded. After quality control, a total of 121,751 SNPs were available for analysis. The researchers used a logistic regression model to test for association between each SNP and AS, adjusting for sex, age, and the first 10 principal components of ancestry. The results were then meta-analyzed across all populations using a fixed-effects model.

The GWAS identified 13 new risk loci and 12 additional AS-associated haplotypes. The most significant association was observed at the HLA-B locus , which has long been known to be associated with AS. In addition, the researchers identified four aminopeptidases involved in

peptide processing before MHC class I presentation, which may have implications for the development of new treatments for AS. The summary statistics data sources for this GWAS are publicly available through the National Human Genome Research Institute (NHGRI) GWAS Catalog. The catalog provides a comprehensive collection of published GWAS summary statistics, including p-values, odds ratios, and effect sizes, for a wide range of complex diseases and traits. The AS GWAS summary statistics data can be accessed through the NHGRI GWAS Catalog website or downloaded directly from the European Genome-phenome Archive (EGA).

In conclusion, the GWAS of AS has identified new genetic variants associated with the disease, shedding new light on the underlying genetic factors contributing to its development. The summary statistics data sources for this study are publicly available, providing a valuable resource for future research on AS and other complex diseases.

GWAS of vitiligo (ViT) by Jin Y et al[11].

Vitiligo is a chronic autoimmune skin disorder characterized by the loss of melanocytes, resulting in depigmented patches on the skin. The genetic basis of vitiligo has been the subject of extensive research, and recent genome-wide association studies (GWAS) have identified numerous susceptibility loci associated with the disease.

The summary statistics data sources used in this GWAS study included three separate datasets: GWAS1, GWAS2, and GWAS3. GWAS1 and GWAS2 were previous linkage and GWAS studies that identified 27 vitiligo susceptibility loci in patients of European ancestry. GWAS3 was a new GWAS study that included augmented controls from GWAS1 and GWAS2, genome-wide imputation, and meta-analysis of all three GWAS datasets. The combined analyses included 4,680 cases and 39,586 controls and identified 23 new loci and 7 suggestive loci associated with vitiligo. The identified loci were found to encode immune and apoptotic regulators, as well as several melanocyte regulators. Bioinformatic analyses indicated a predominance of causal regulatory variation, some corresponding to expression quantitative trait loci (eQTL) at these loci. The identified genes provide a framework for vitiligo genetic architecture and pathobiology, highlighting relationships to other autoimmune diseases and melanoma, and offering potential targets for treatment.

The study also included an independent replication study that included 1827 unrelated European vitiligo cases and 2181 unrelated European controls not included in any of the GWAS datasets. All subjects provided written informed consent, and the study was carried out under the jurisdiction of each local institutional review board with overall oversight of the Colorado Multiple Institutional Review Board (COMIRB). While this study provides valuable insights into the genetic basis of vitiligo, there are some limitations to consider when interpreting the results. For example, the study only included patients of European ancestry, and further research is needed to determine if the identified loci are also associated with vitiligo in other populations. Additionally, the study did not investigate the functional consequences of the identified variants, and further research is needed to determine how these variants contribute to the development of vitiligo.

Overall, this GWAS study provides important new insights into the genetic basis of vitiligo and offers potential targets for the development of new treatments for this chronic autoimmune skin

disorder.

GWAS of bronchiectasis (BE) from FinnGen (R9) Consortium[12].

Bronchiectasis is defined by the International ICD-10 and according to the FinnGen Consortium's J10_BRONCHIECTASIS control, total number of diseases 2597 (women 1433 men 1164), median age at first event (years): 63.45, for details see https://risteys.finregistry.fi /endpoints/J10_BRONCHIECTASIS. Summary-level GWAS data for bronchiectasis are from the latest R9 version of the FInnGen consortium, detailed information is available at https://www.finngen.fi/en/access_results

Intermediary phenotype

GWAS of Triglyceride, Low Density Lipoprotein Cholesterol and High Density Lipoprotein Cholesterol by Cristen J Willer *et al*[13].

The GWAS study analyzed in this article aimed to identify new loci and refine known loci associated with lipid levels, including low-density lipoprotein (LDL) cholesterol, high-density lipoprotein (HDL) cholesterol, triglycerides, and total cholesterol. The study examined 188,578 individuals using genome-wide and custom genotyping arrays and identified and annotated 157 loci associated with lipid levels at $P < 5\times10^{-8}$, including 62 loci not previously associated with lipid levels in humans. Of the 62 novel loci, 24 demonstrated the strongest evidence of association with HDL cholesterol, 15 with LDL cholesterol, 8 with triglyceride levels, and 15 with total cholesterol. The effects of newly identified loci were generally smaller than in earlier GWAS. For the 62 newly identified variants, trait variance explained in the Framingham offspring were 1.6% for HDL cholesterol, 2.1% for triglycerides, 2.4% for LDL cholesterol, and 2.6% for total cholesterol .

To investigate connections between the new loci and known lipid biology, the study catalogued genes within 100 kb of the peak associated SNPs and searched PubMed and OMIM for occurrences of these gene names and their aliases in the context of relevant keywords. After manual curation, the study identified at least one strong candidate in 32 of the 62 loci (52%). For the remaining 30 loci, the study found no literature support for the role of a nearby gene on blood lipid levels . Among independent variants ($r^2 < 0.1$) with $P < 0.1$ in the GWAS-only analysis, a significant excess were concordant in direction of effect for HDL (62.9% in 1,847 SNPs, $P < 10^{-16}$), LDL (58.6% of 1,730 SNPs, $P < 10^{-16}$), triglyceride levels (59.1% of 1,783 SNPs, $P < 10^{-16}$), and total cholesterol (61.0% of 1,904 SNPs, $P < 10^{-16}$), suggesting many additional loci to be discovered in future studies .

GWAS of Body Mass Index (BMI) by GIANT consortium[14].

The summary statistics data sources for the genome-wide association study (GWAS) of Body Mass Index (BMI) were obtained from two previous studies: Wood et al. and Locke et al. Before conducting the meta-analysis with the UK Biobank (UKB) data, the researchers filtered out SNPs that did not match the pairs of alleles in the HRS and UKB and those that had reported allele frequencies that were too different from that calculated using unrelated participants of HRS. After filtering the data, the researchers performed a fixed-effect inverse variance weighted meta-analysis using the software METAL . This approach allowed them to combine the summary

statistics from the two previous studies with the GWAS of height and BMI performed in ~450 000 UK Biobank participants of European ancestry.

The combined GWAS meta-analysis reached N ~700 000 individuals and substantially increased the number of GWAS signals associated with BMI. The researchers identified 941 near-independent SNPs associated with BMI at a revised genome-wide significance threshold of $P < 1 \times 10^{-8}$, including 751 BMI-associated SNPs located within loci not previously identified by these two GWAS. The near-independent genome-wide significant SNPs explained ~6.0% of the variance of BMI in an independent sample from the Health and Retirement Study (HRS). This finding suggests that there are likely many more genetic factors that influence BMI that have yet to be identified.

To control for potential confounding variables, the researchers performed LDSC to quantify the level of confounding in GWAS due to population stratification as well as quantifying the genetic correlation between BMI and other traits. This approach allowed them to identify potential confounding variables and control for them in their analysis.

GWAS of 25-hydroxyvitamin D (25OHD) by Manousaki D et al[15].
The study used imputed genotypes from 401,460 white British UK Biobank participants with available 25OHD levels, retaining single-nucleotide polymorphisms (SNPs) with minor allele frequency (MAF) > 0.1% and imputation quality score > 0.3. The summary statistics data sources for this study include a Manhattan plot, a quantile-quantile (QQ) plot, and a list of 69 independent loci that contribute to serum 25OHD levels. The Manhattan plot displays the genome-wide association of 25OHD graphed by chromosome positions and -log10 P value. The QQ plot shows the distribution of observed P values compared to the expected distribution under the null hypothesis of no association.

The list of 69 independent loci that contribute to serum 25OHD levels was identified through a linear mixed model GWAS on standardized log-transformed 25OHD, adjusting for age, sex, season of measurement, and vitamin D supplementation. The loci were identified based on a threshold of genome-wide significance (P < 5 x 10^-8) and were independent of previously reported loci. In addition to the 69 independent loci, the study also identified 12 SNPs that achieved significant interaction p values. The direction of the beta for the interaction term genotype*season summer was in the same direction as the direction of the beta on 25OHD levels, meaning that the vitamin D lowering effect of these SNPs 'blunts' the expected increase in 25OHD in summer.

The study also conducted in silico functional follow-up gene prioritization and enrichment analyses. Gene prioritization analysis suggested 70 genes with false discovery rate (FDR) < 5% which might plausibly underlie the distribution of association statistics seen in the single variant results. At many loci, genes within the vitamin D metabolism pathway were suggested as plausible candidates. For example, DEPICT prioritized DHCR7 at the study.

GWAS of smoking by GSCAN[16].

The study aimed to identify genetic variants associated with smoking behavior and related phenotypes. The data sources used in the study were collected from multiple studies and included both related and unrelated individuals. The summary statistics data sources for smoking included GWAS meta-analyses of five substance use phenotypes. These phenotypes were smoking initiation, smoking cessation, age of smoking initiation, cigarettes per day, and drinks per week. The data sources were obtained from studies that genotyped participants on genome-wide arrays and imputed their genotypes to the Haplotype Reference Consortium using either Minimac3 or IMPUTE2.

The studies used RVTESTS to generate GWAS summary statistics for each sample. For studies composed primarily of related individuals, covariates including genetic principal components were regressed out under a linear model. The residuals were then inverse-normalized (except for 23andMe) and tested for an additive effect of each variant under a linear mixed model with a genetic kinship matrix. Family studies followed this analysis for all phenotypes, even binary phenotypes such as smoking initiation and cessation. For studies of entirely unrelated individuals, the same analysis was followed for quasi-continuous phenotypes (AgeSmk, CigDay, DrnkWk). However, for binary phenotypes (SmkInit and SmkCes), additive genetic effects were estimated under a logistic model. The GWAS summary statistics data sources for smoking can be downloaded from the world wide web. Association results for all SNPs that passed quality-control filters in a GWAS meta-analysis of each of the five substance use phenotypes are provided. However, the research participants from 23andMe are excluded from the data.

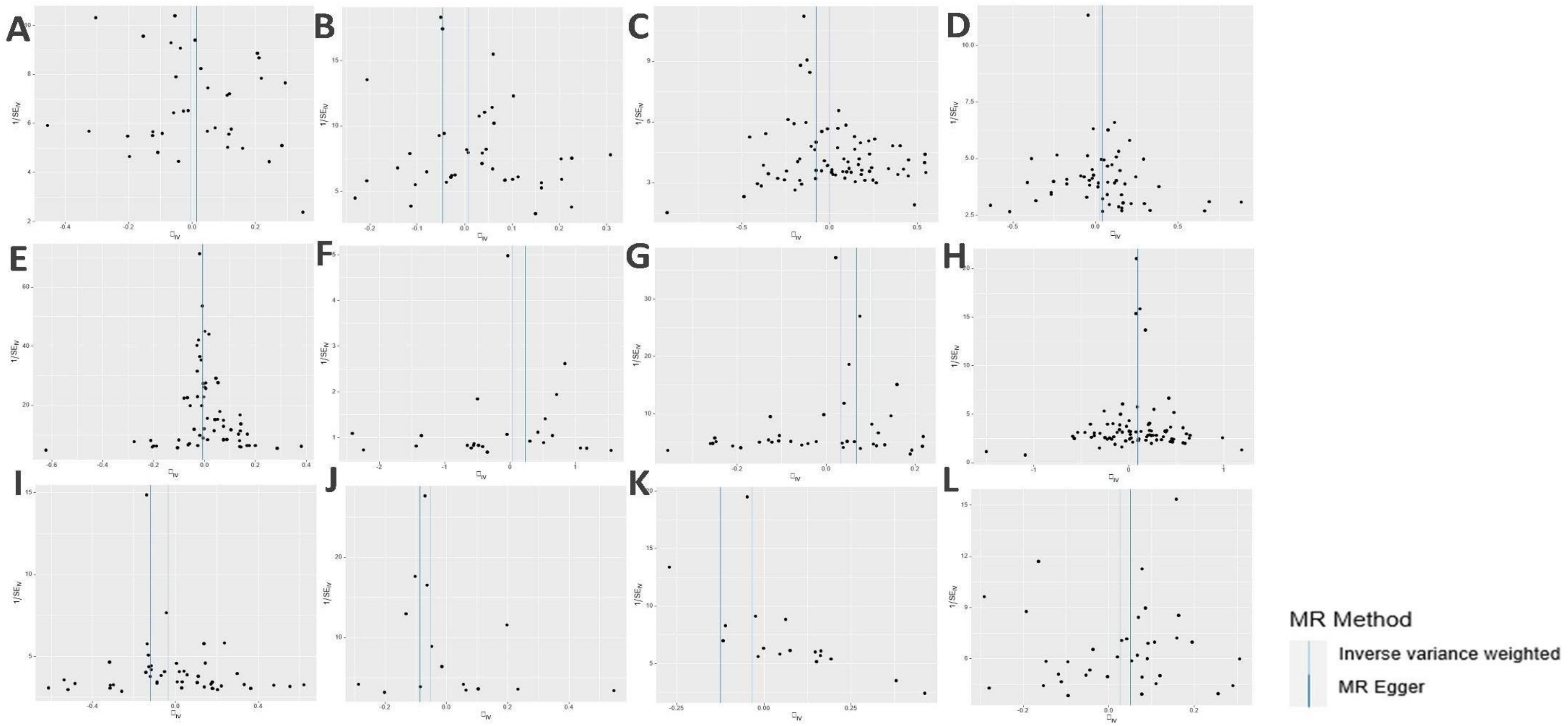

Supplementary Fig. 1 Funnel plot of instrument precision against instrumental variable estimates for each genetic variant separately for Mendelian randomization analysis of autoimmune disease on bronchiectasis risk. (A)Vitiligo(B)Systemic lupus erythematosus(C)Crohn's disease (D)Ulcerative colitis(E)Psoriasis (F)Ankylosing spondylitis(G)Type 1 diabetes (H)Rheumatoid arthritis(I)Multiple sclerosis (J)Celiac disease(K)Primary sclerosing cholangitis(I)Primary biliary cirrhosis. Solid vertical line is the (random- effect) inverse-variance weighted estimate.

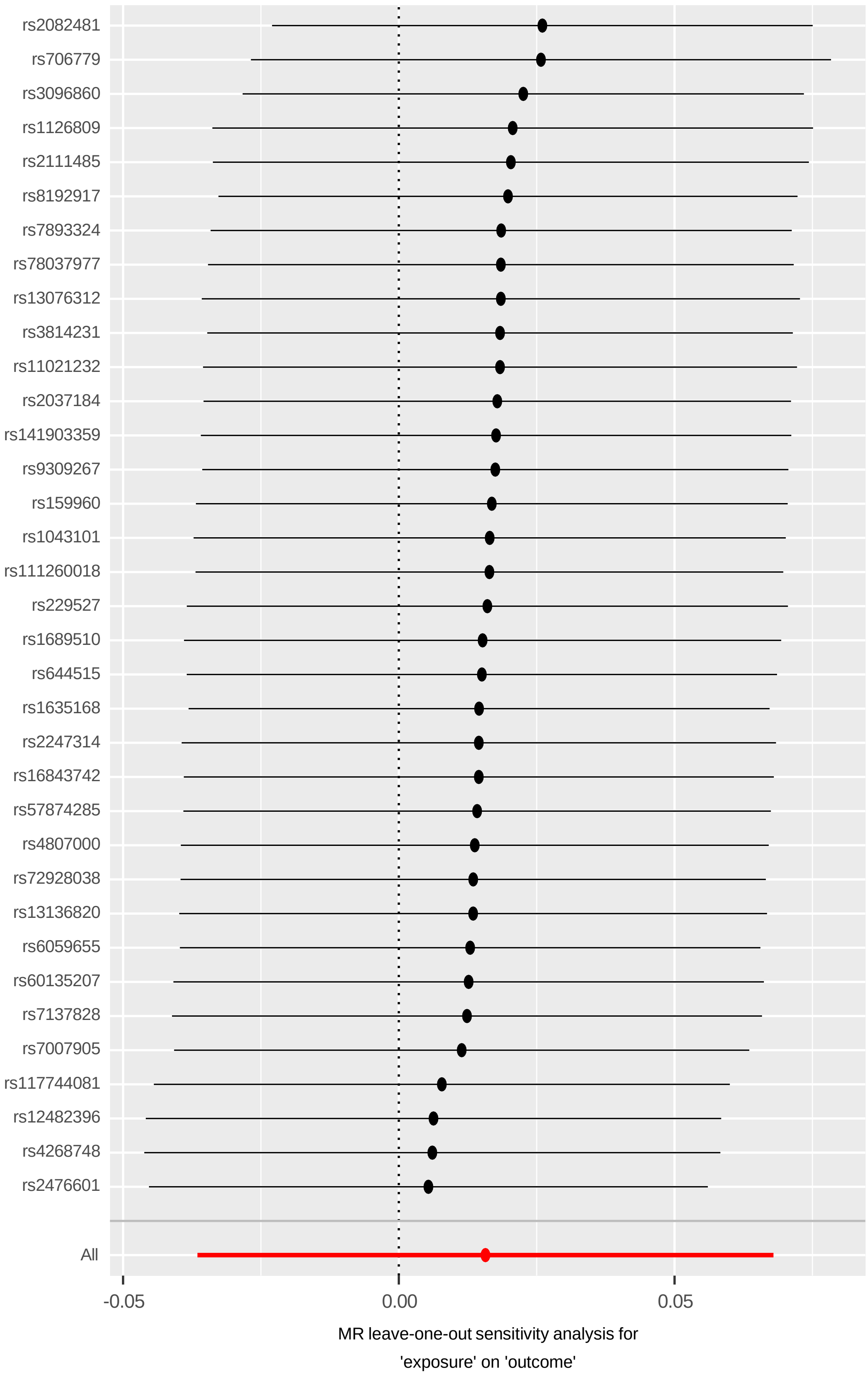

Supplementary Fig. 2 Leave-one-out plot for MR analysis of Vitiligo on bronchiectasis risk

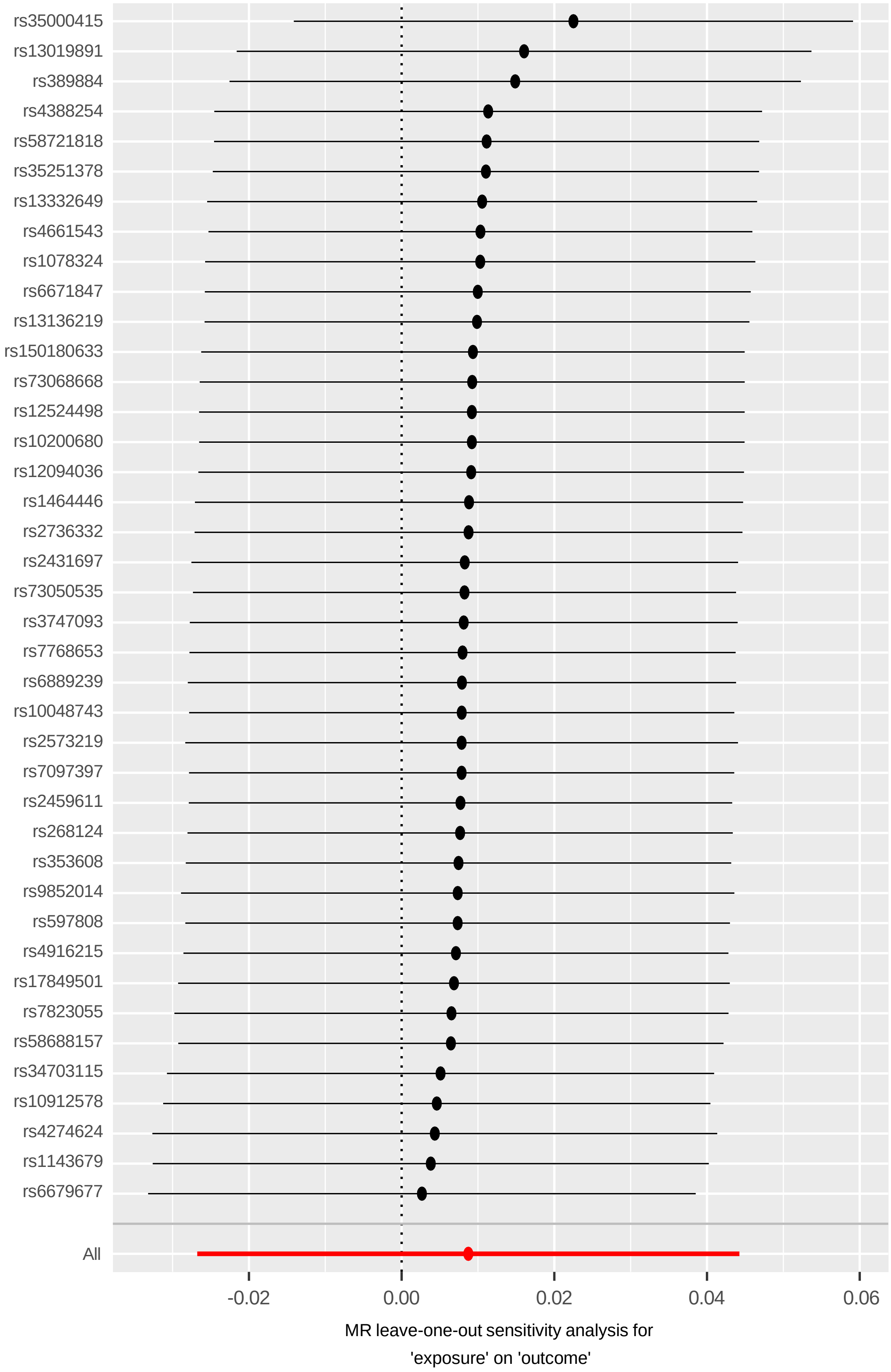

Supplementary Fig. 3 Leave-one-out plot for MR analysis of Systemic lupus erythematosus on bronchiectasis risk

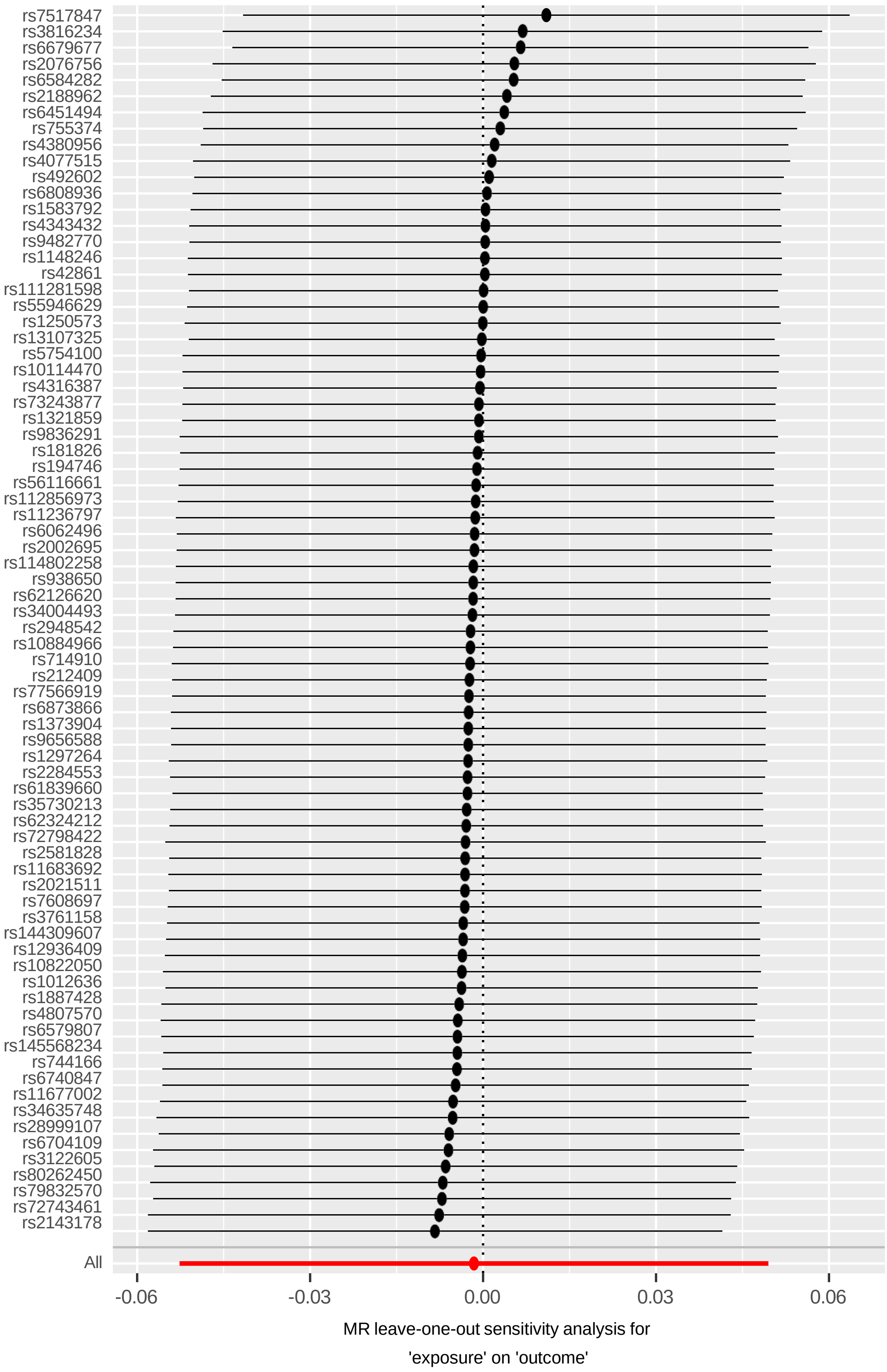

Supplementary Fig. 4 Leave-one-out plot for MR analysis of Crohn's disease on bronchiectasis risk

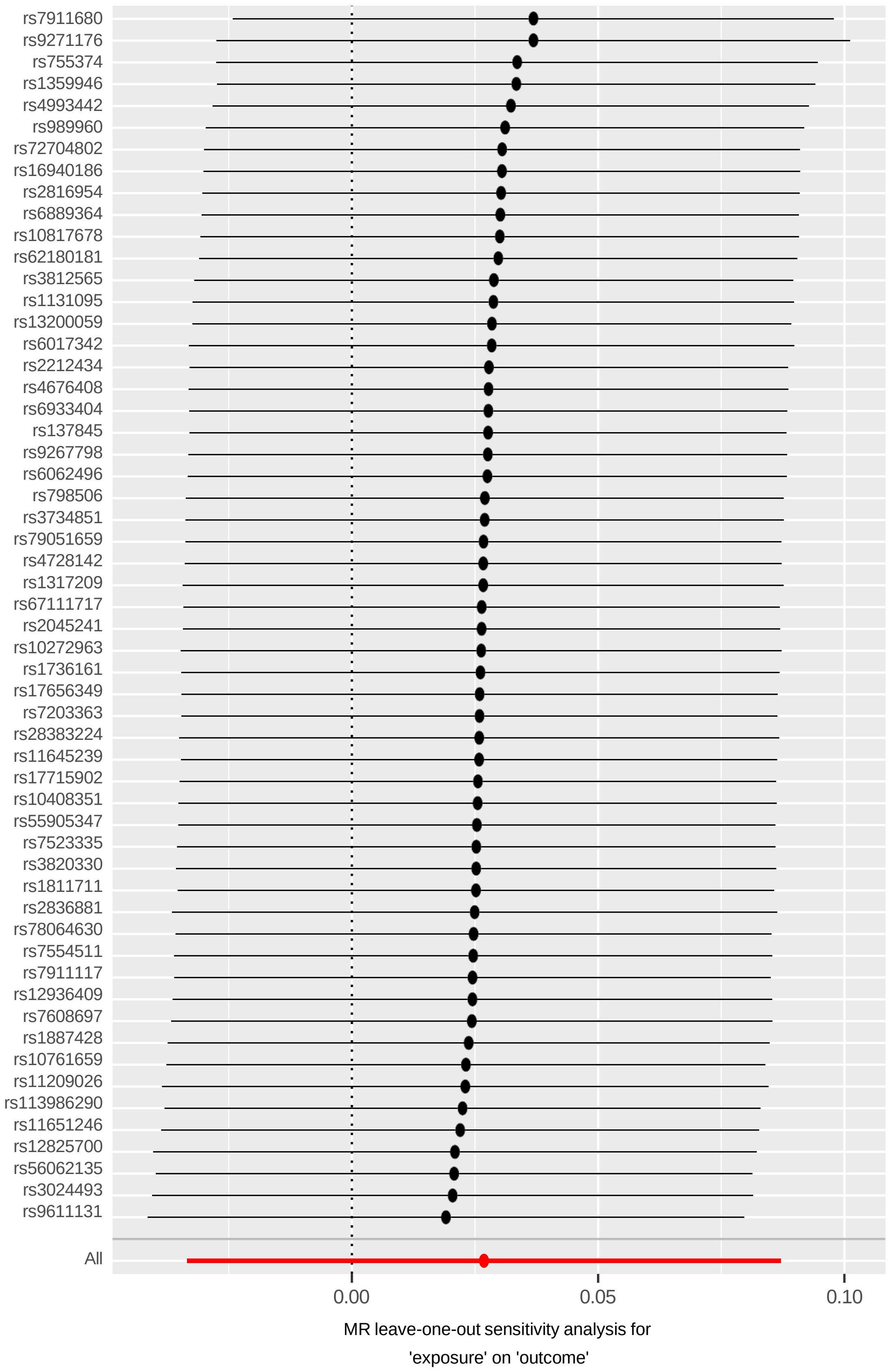

Supplementary Fig. 5 Leave-one-out plot for MR analysis of Ulcerative colitis on bronchiectasis risk

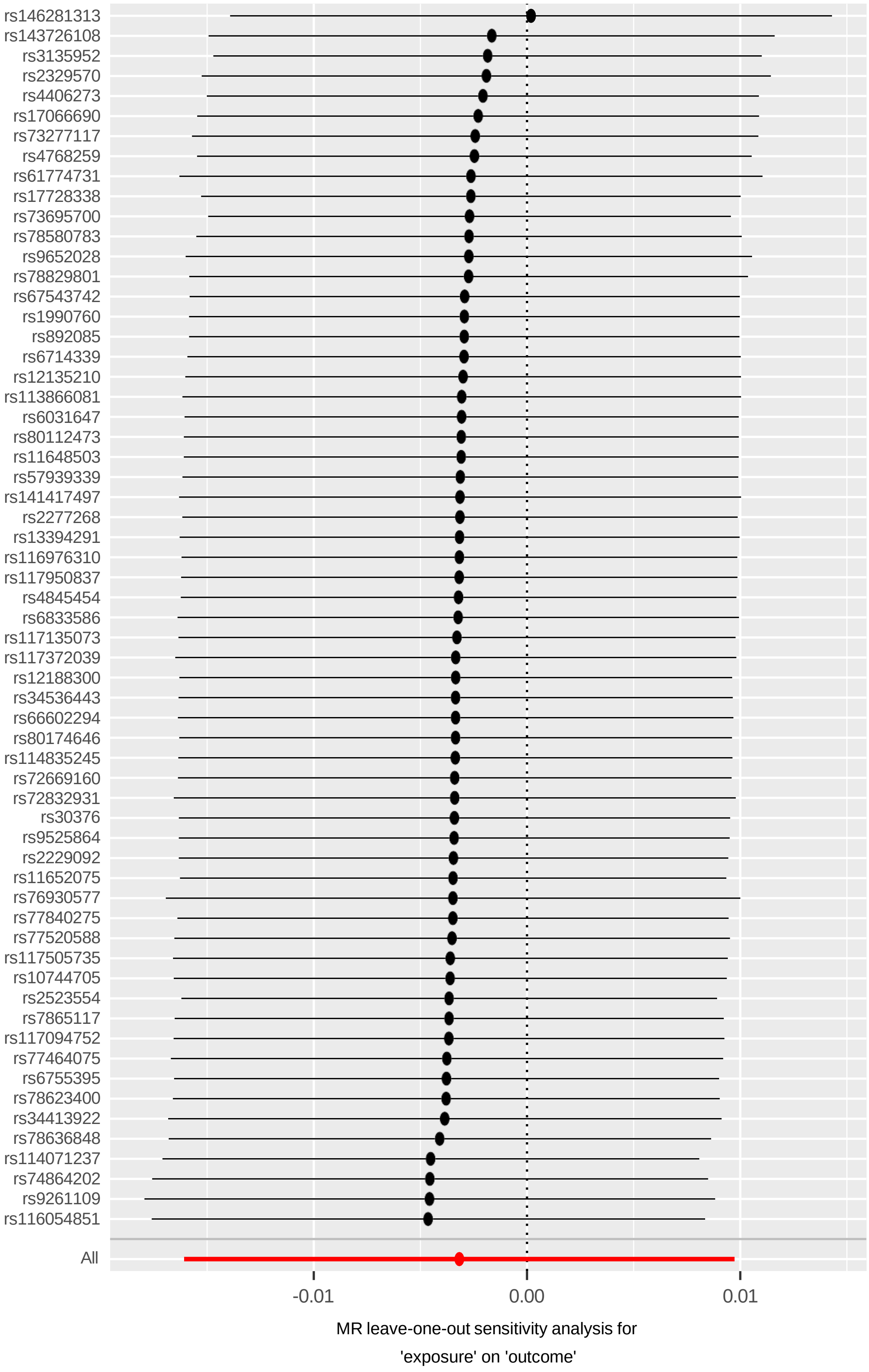

Supplementary Fig. 6 Leave-one-out plot for MR analysis of Psoriasis on bronchiectasis risk

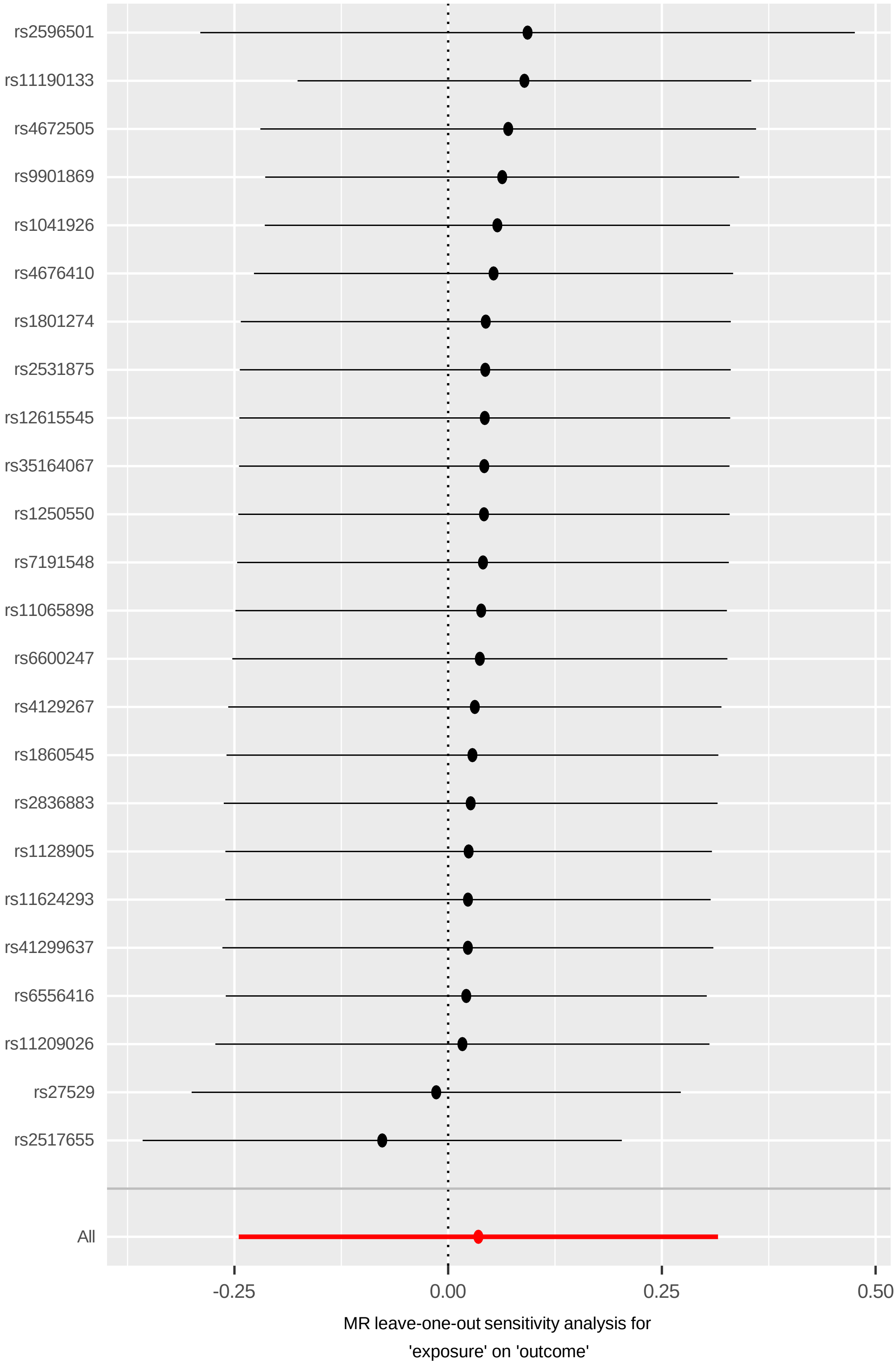

Supplementary Fig. 7 Leave-one-out plot for MR analysis of Ankylosing spondylitis on bronchiectasis risk

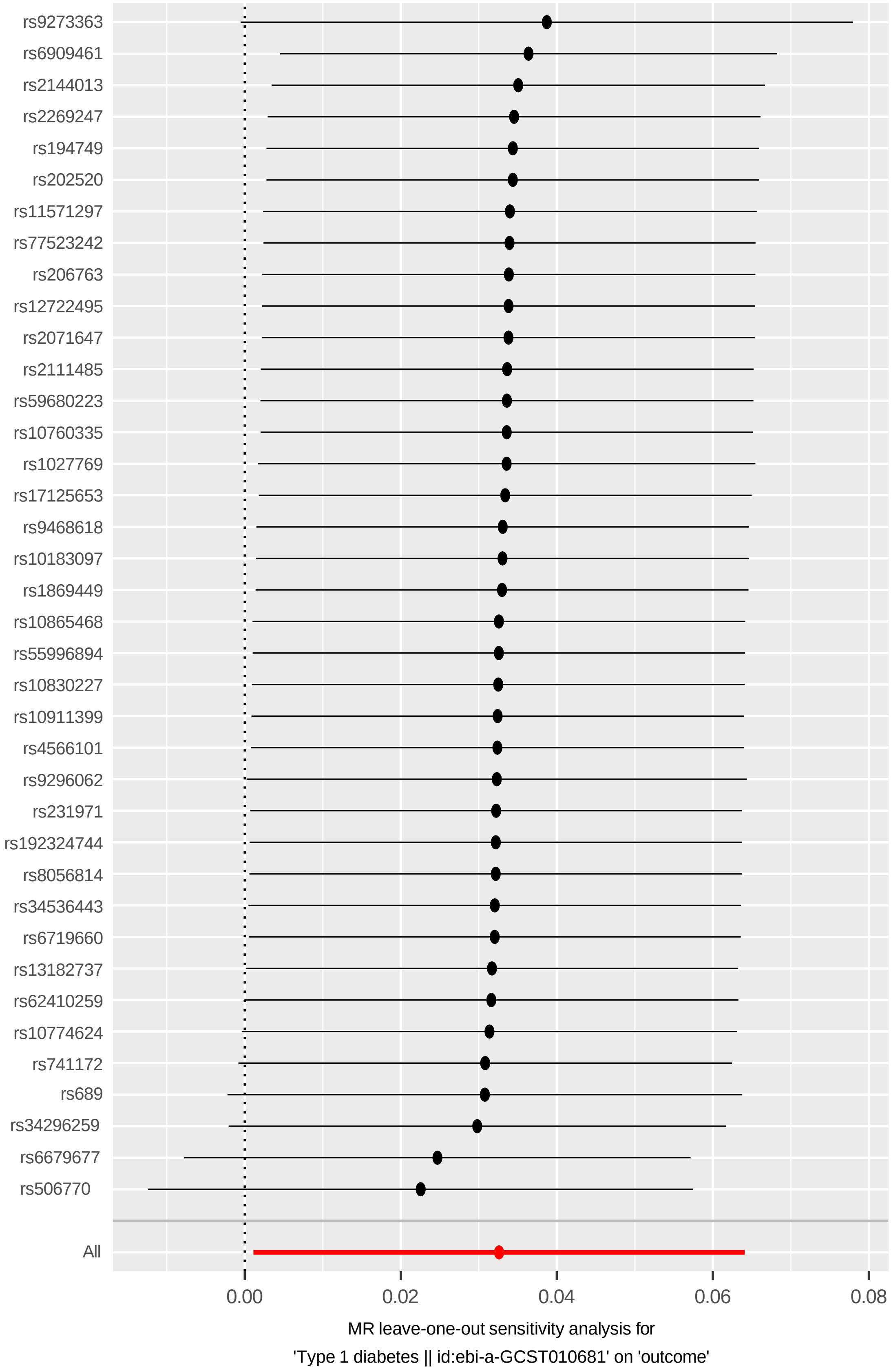

Supplementary Fig. 8 Leave-one-out plot for MR analysis of Type 1 diabetes on bronchiectasis risk

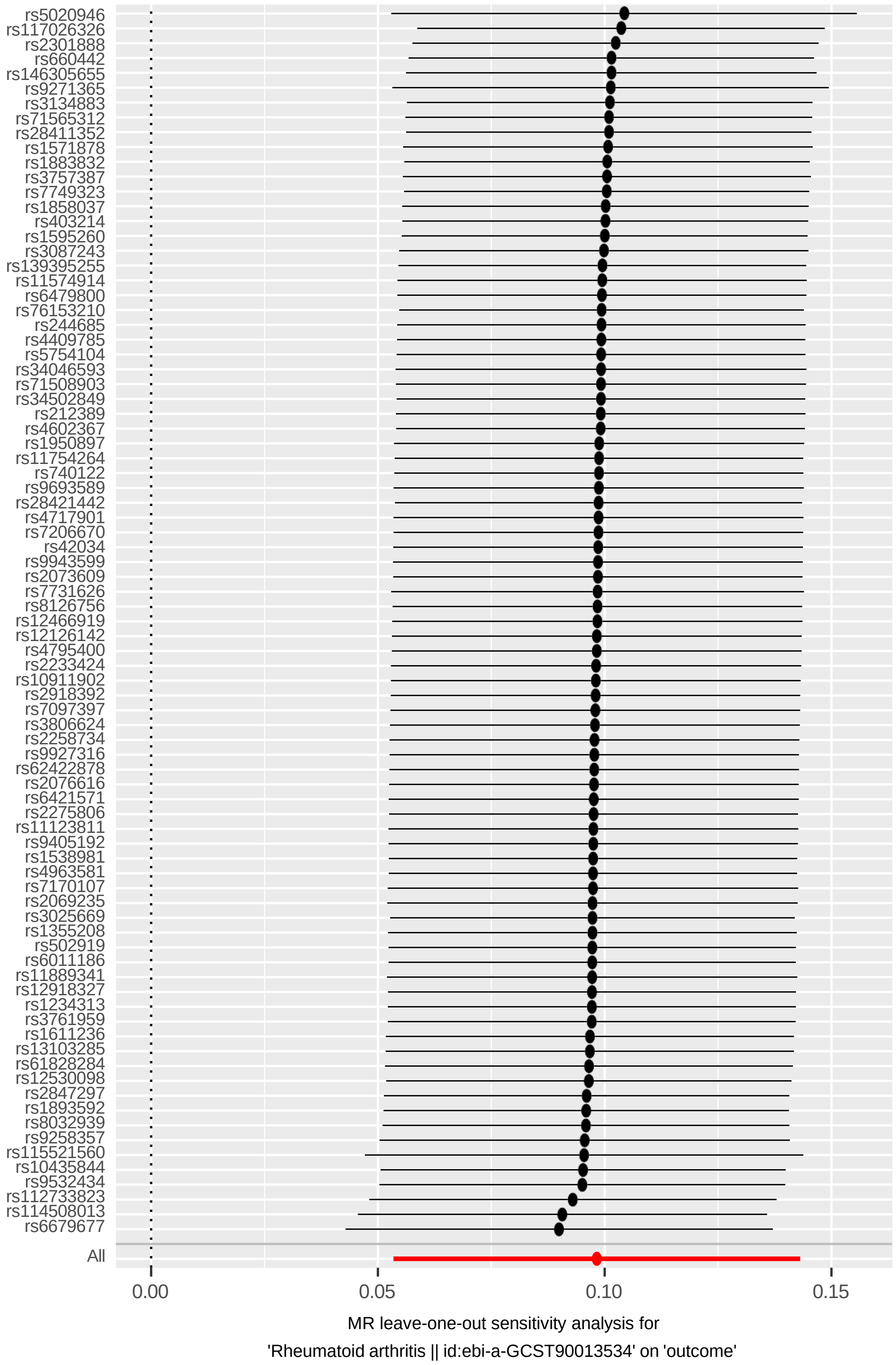

Supplementary Fig. 9 Leave-one-out plot for MR analysis of Rheumatoid arthritis on bronchiectasis risk

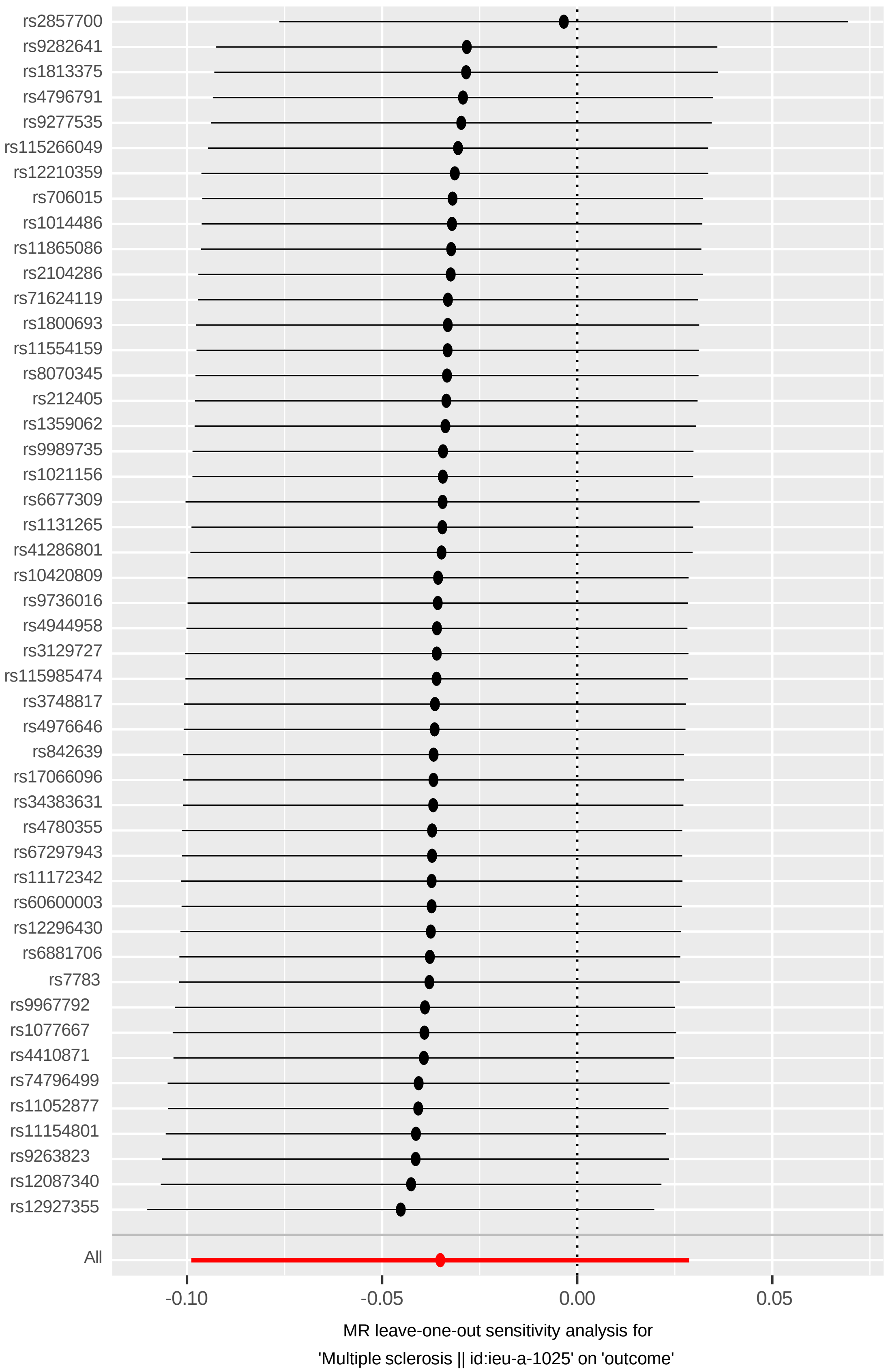

Supplementary Fig. 10 Leave-one-out plot for MR analysis of Multiple sclerosis on bronchiectasis risk

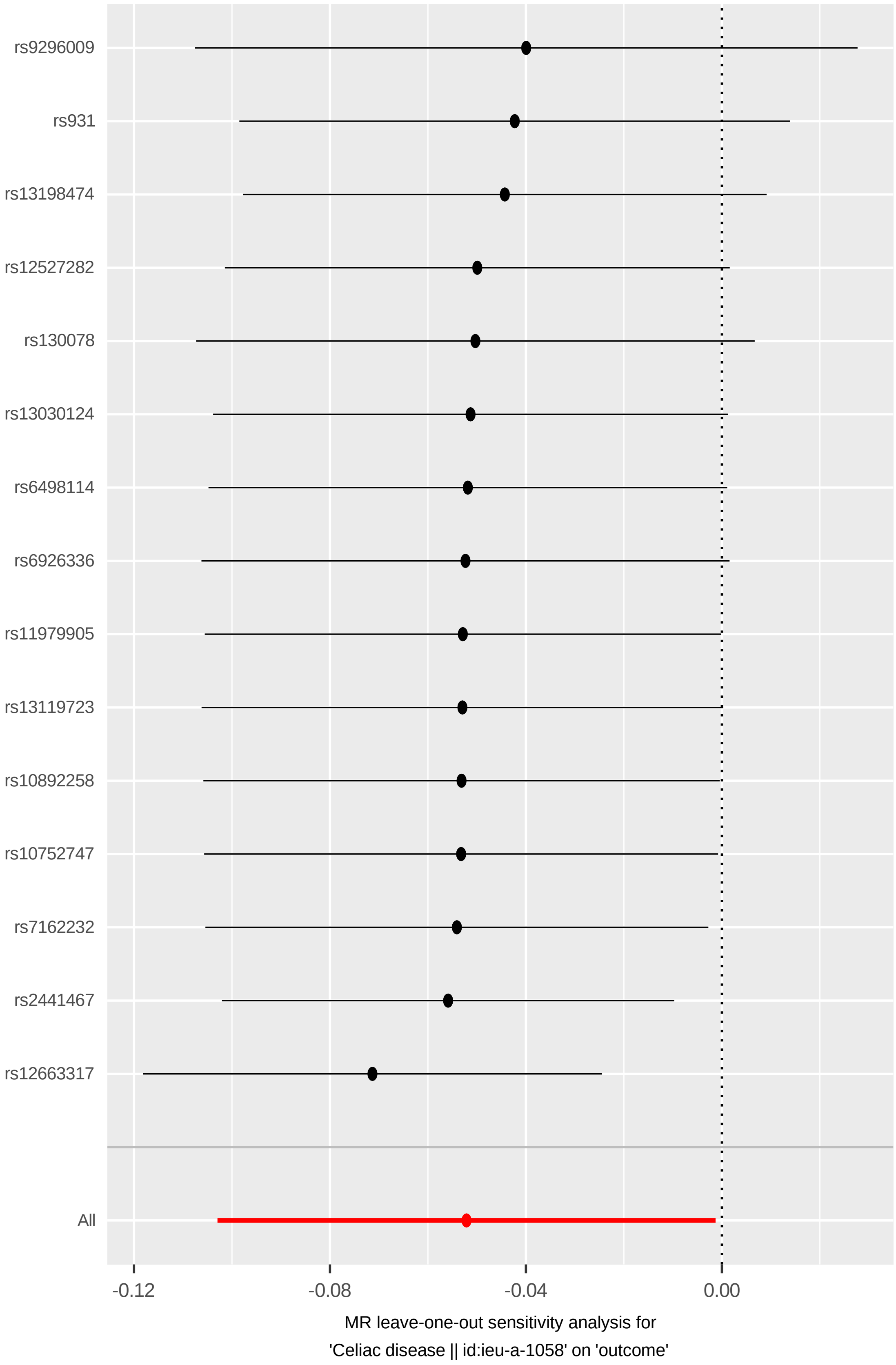

Supplementary Fig. 11 Leave-one-out plot for MR analysis of Celiac disease on bronchiectasis risk

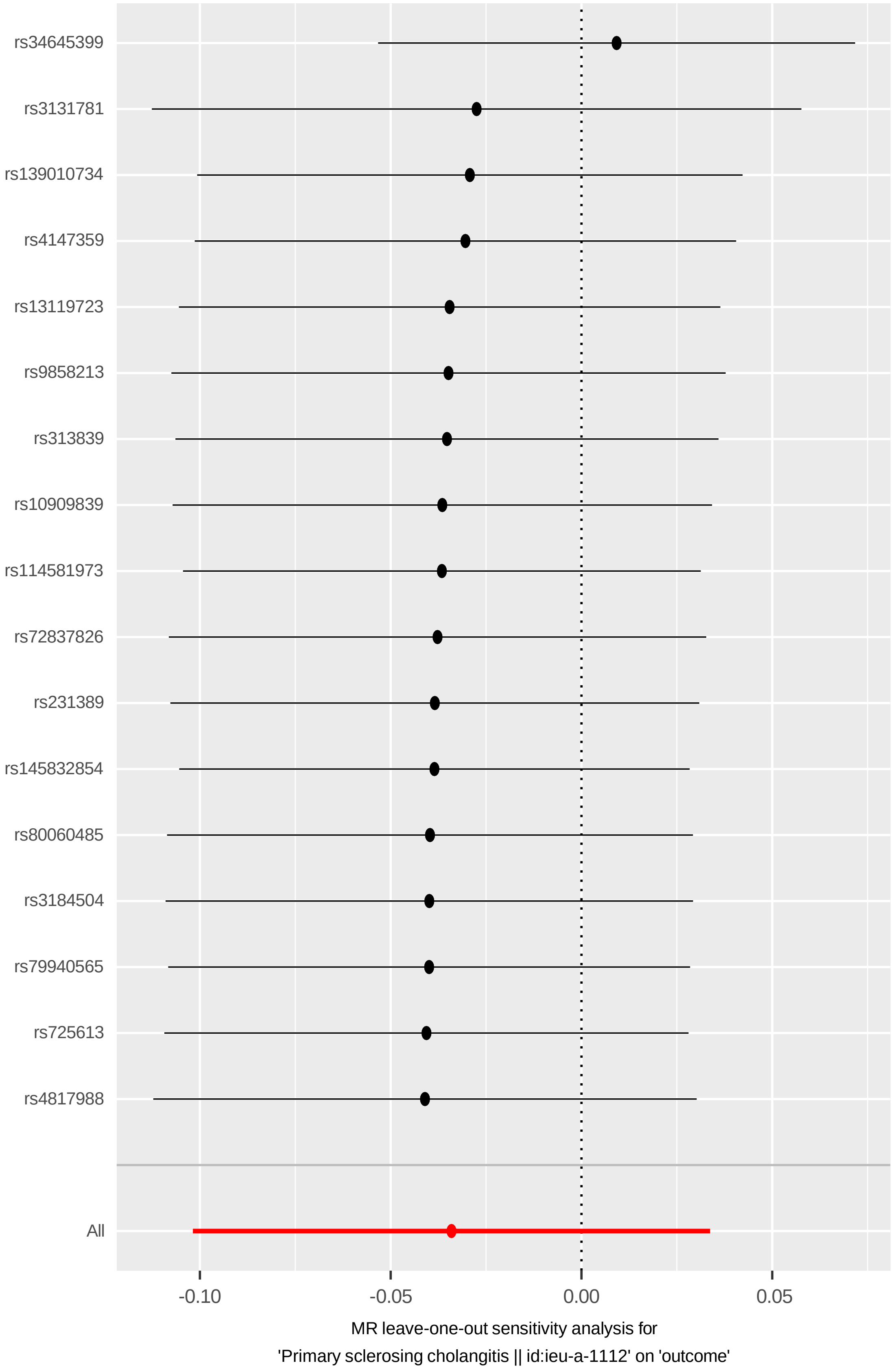

Supplementary Fig. 12 Leave-one-out plot for MR analysis of Primary sclerosing cholangitis on bronchiectasis risk

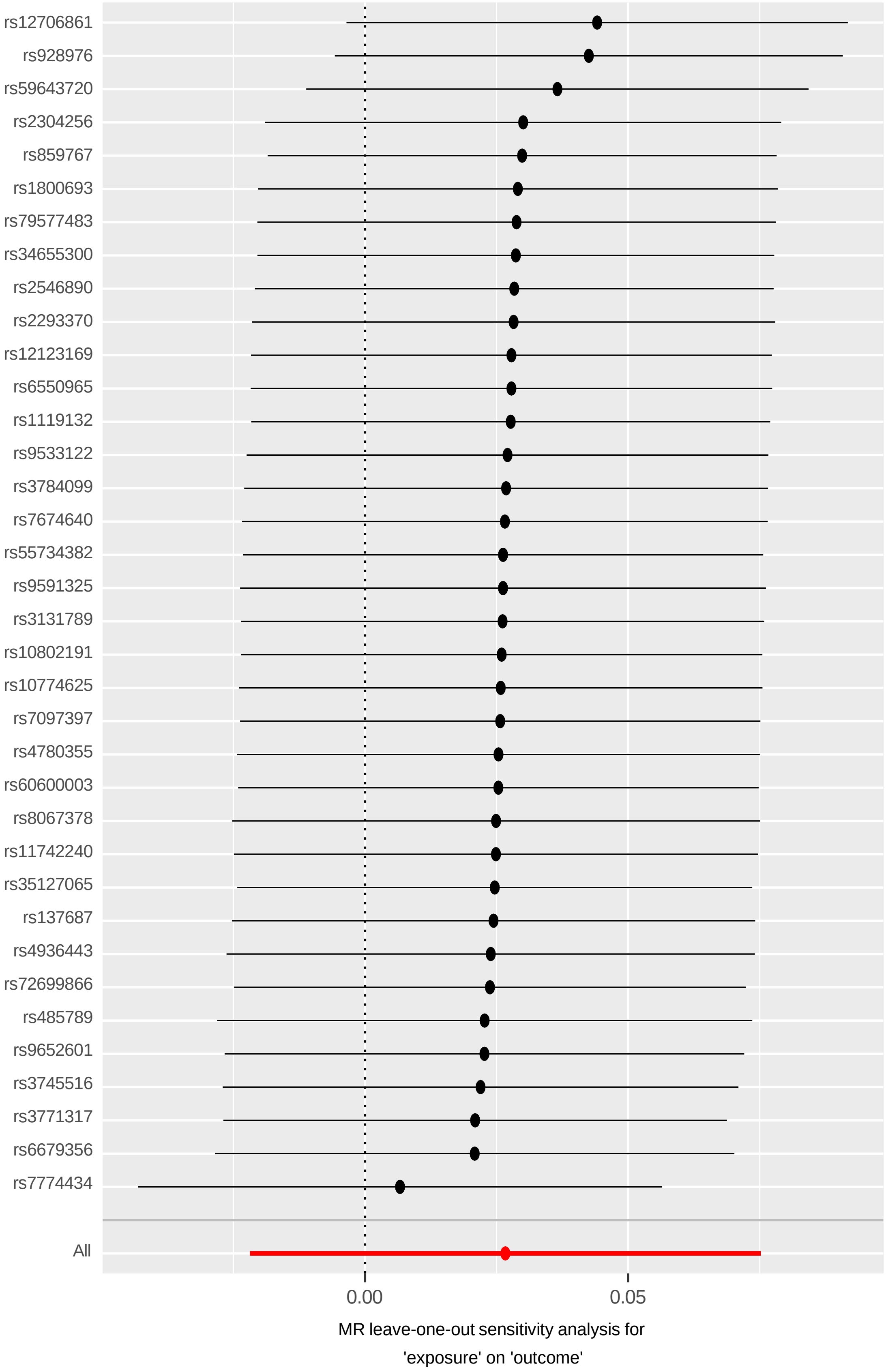

Supplementary Fig. 13 Leave-one-out plot for MR analysis of Primary biliary cirrhosis on bronchiectasis risk

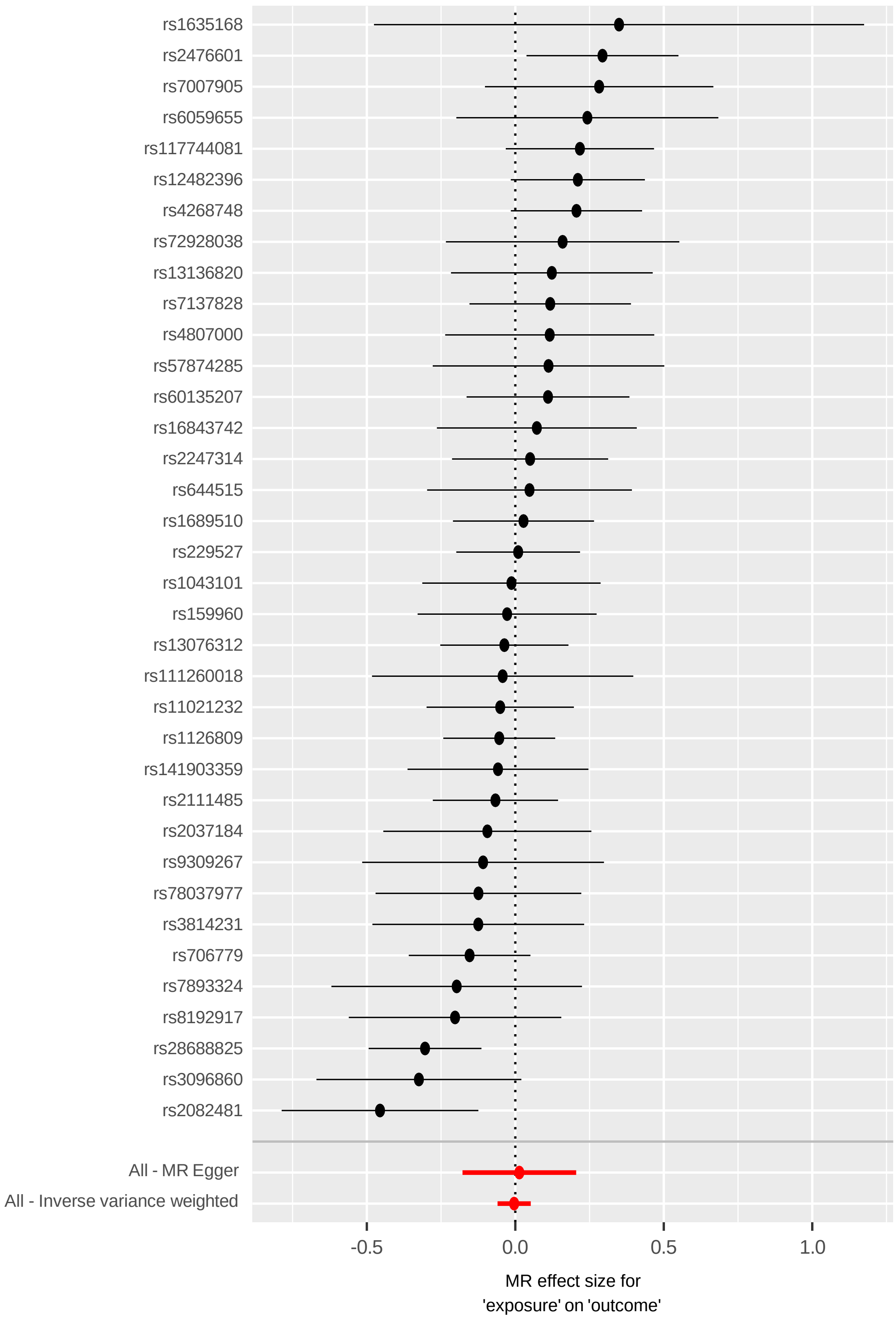

Supplementary Fig. 14 Single-SNP analysis forest plots of the effect of Vitiligo on bronchiectasis phenotypes. Point estimates represent the variant-specific ratio estimates for each SNP (in black), and the inverse-variance weighted (IVW) estimate (in red). Horizontal lines represent 95% confidence intervals around the variant-specific ratio estimates and the IVW estimate.

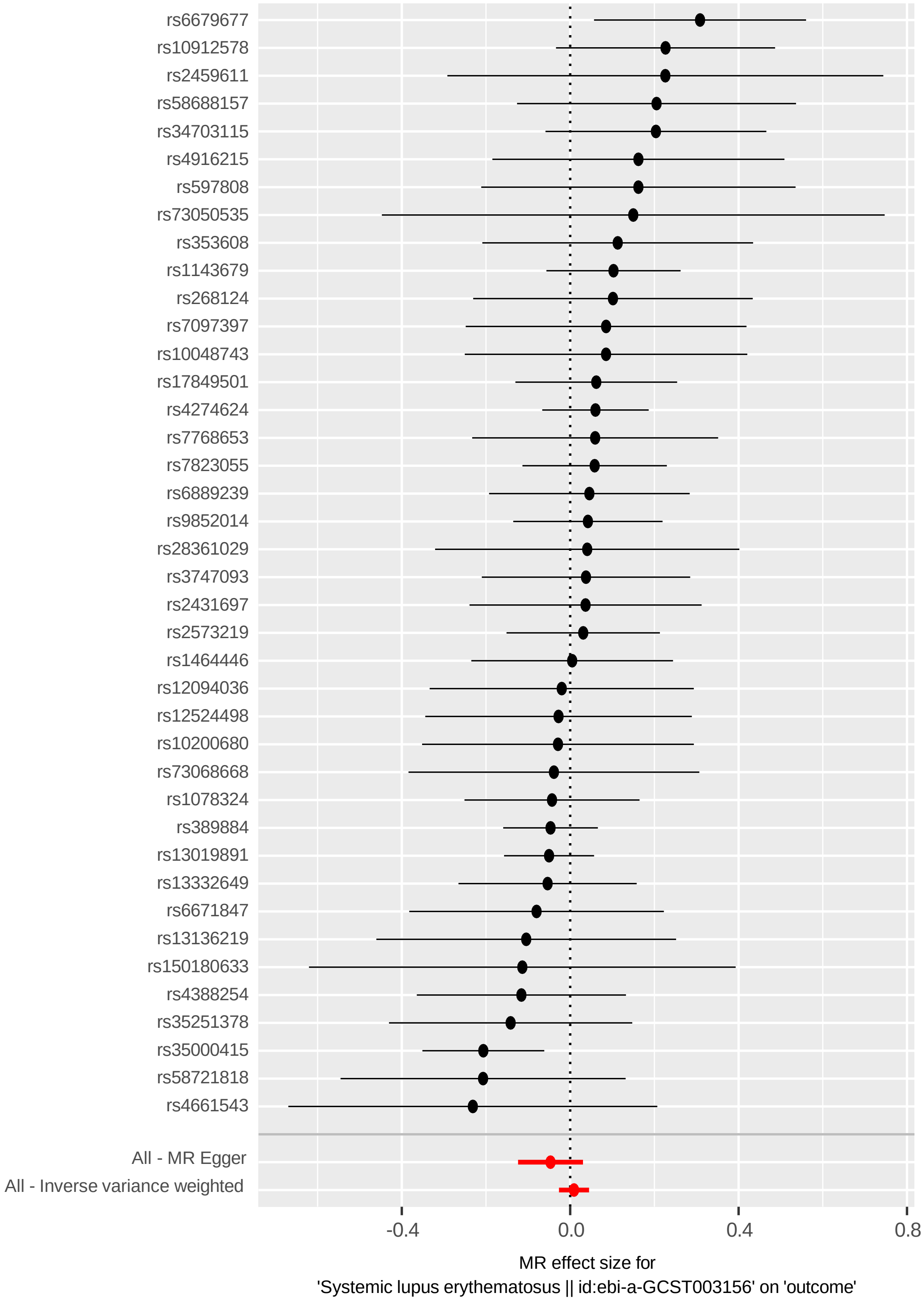

Supplementary Fig. 15 Single-SNP analysis forest plots of the effect of Systemic lupus erythematosus on bronchiectasis phenotypes. Point estimates represent the variant-specific ratio estimates for each SNP (in black), and the inverse-variance weighted (IVW) estimate (in red). Horizontal lines represent 95% confidence intervals around the variant-specific ratio estimates and the IVW estimate.

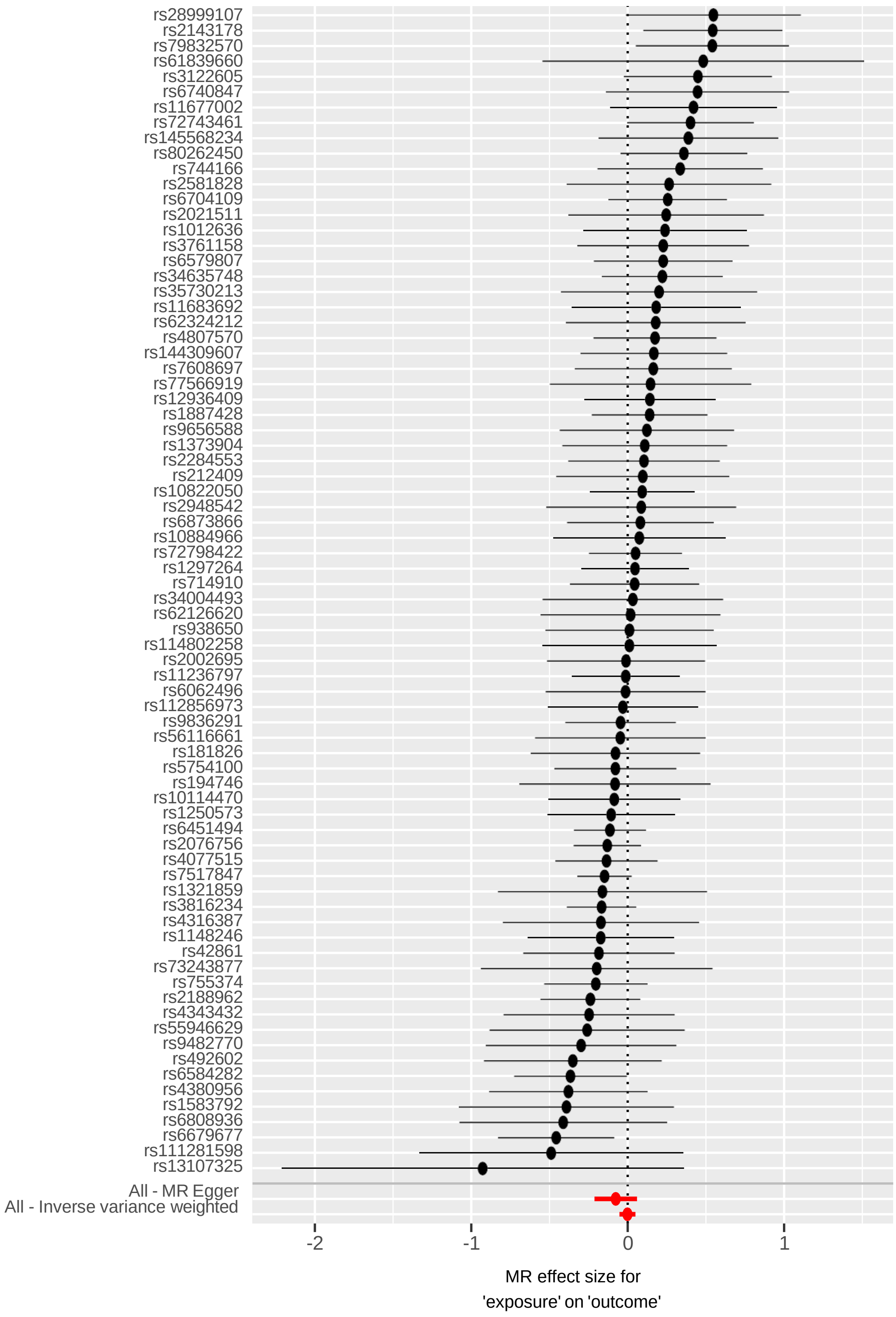

Supplementary Fig. 16 Single-SNP analysis forest plots of the effect of Crohn's disease on bronchiectasis phenotypes. Point estimates represent the variant-specific ratio estimates for each SNP (in black), and the inverse-variance weighted (IVW) estimate (in red). Horizontal lines represent 95% confidence intervals around the variant-specific ratio estimates and the

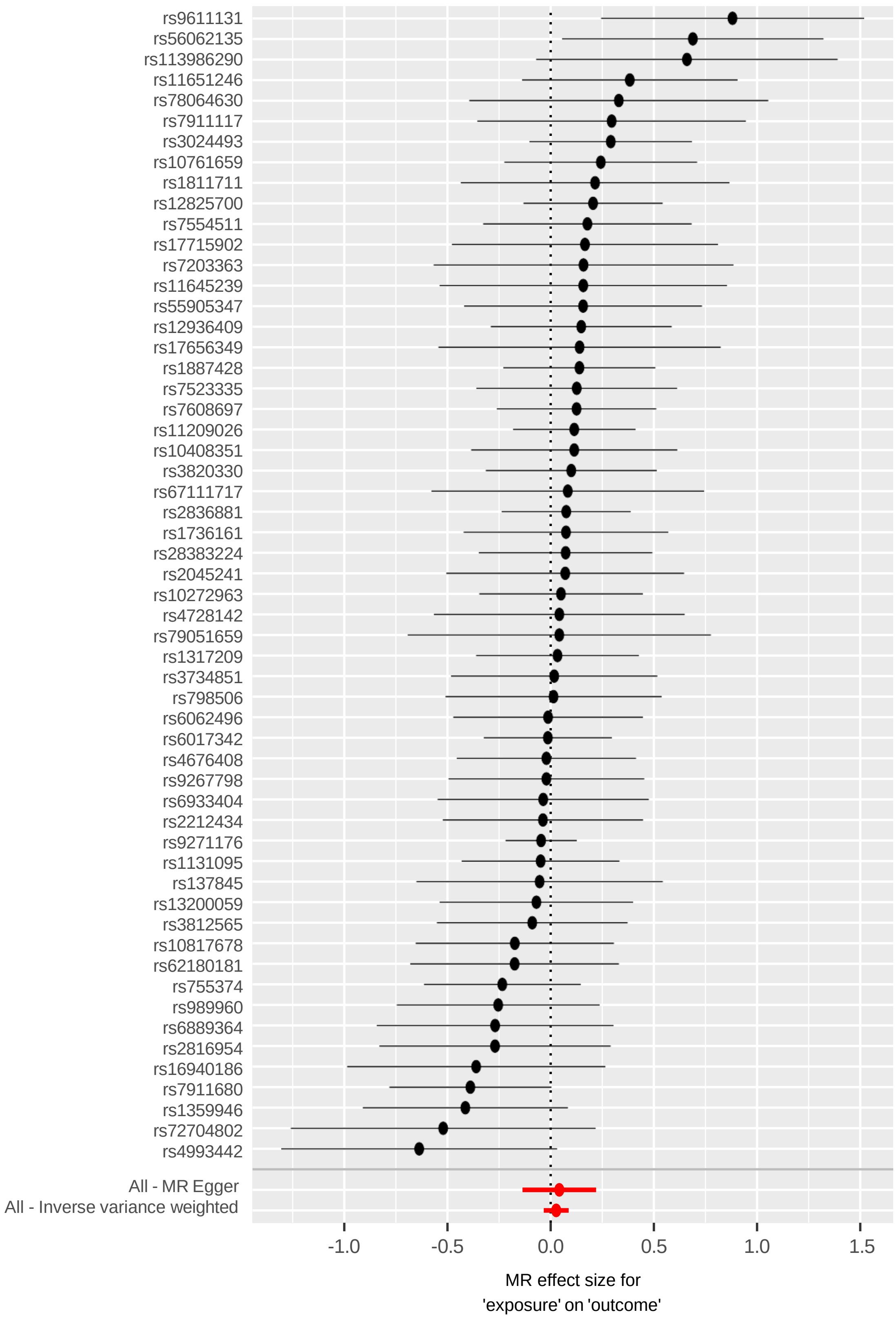

Supplementary Fig. 17 Single-SNP analysis forest plots of the effect of Ulcerative colitis on bronchiectasis phenotypes. Point estimates represent the variant-specific ratio estimates for each SNP (in black), and the inverse-variance weighted (IVW) estimate (in red). Horizontal lines represent 95% confidence intervals around the variant-specific ratio estimates and the

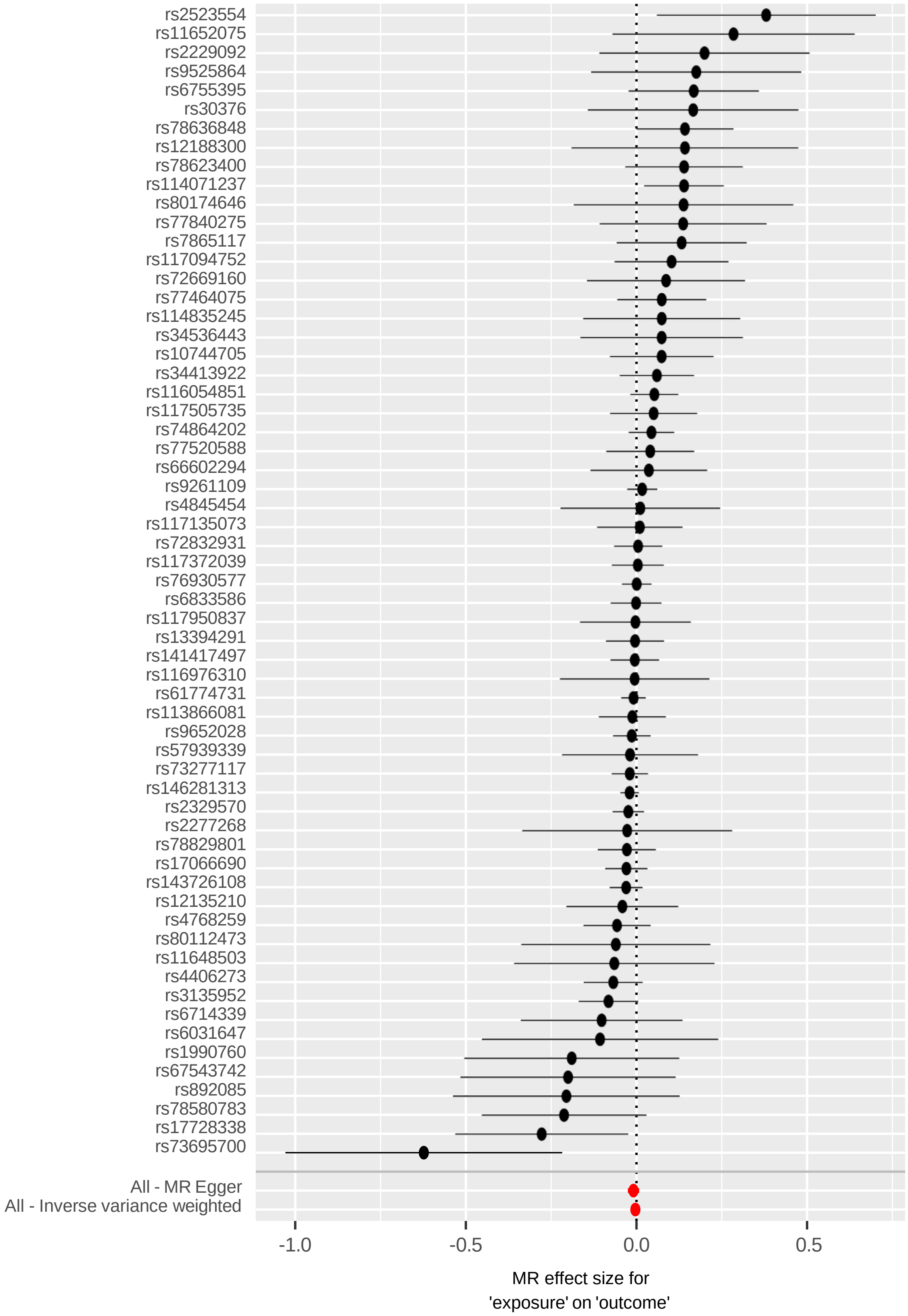

Supplementary Fig. 18 Single-SNP analysis forest plots of the effect of Psoriasis on bronchiectasis phenotypes. Point estimates represent the variant-specific ratio estimates for each SNP (in black), and the inverse-variance weighted (IVW) estimate (in red). Horizontal lines represent 95% confidence intervals around the variant-specific ratio estimates and the

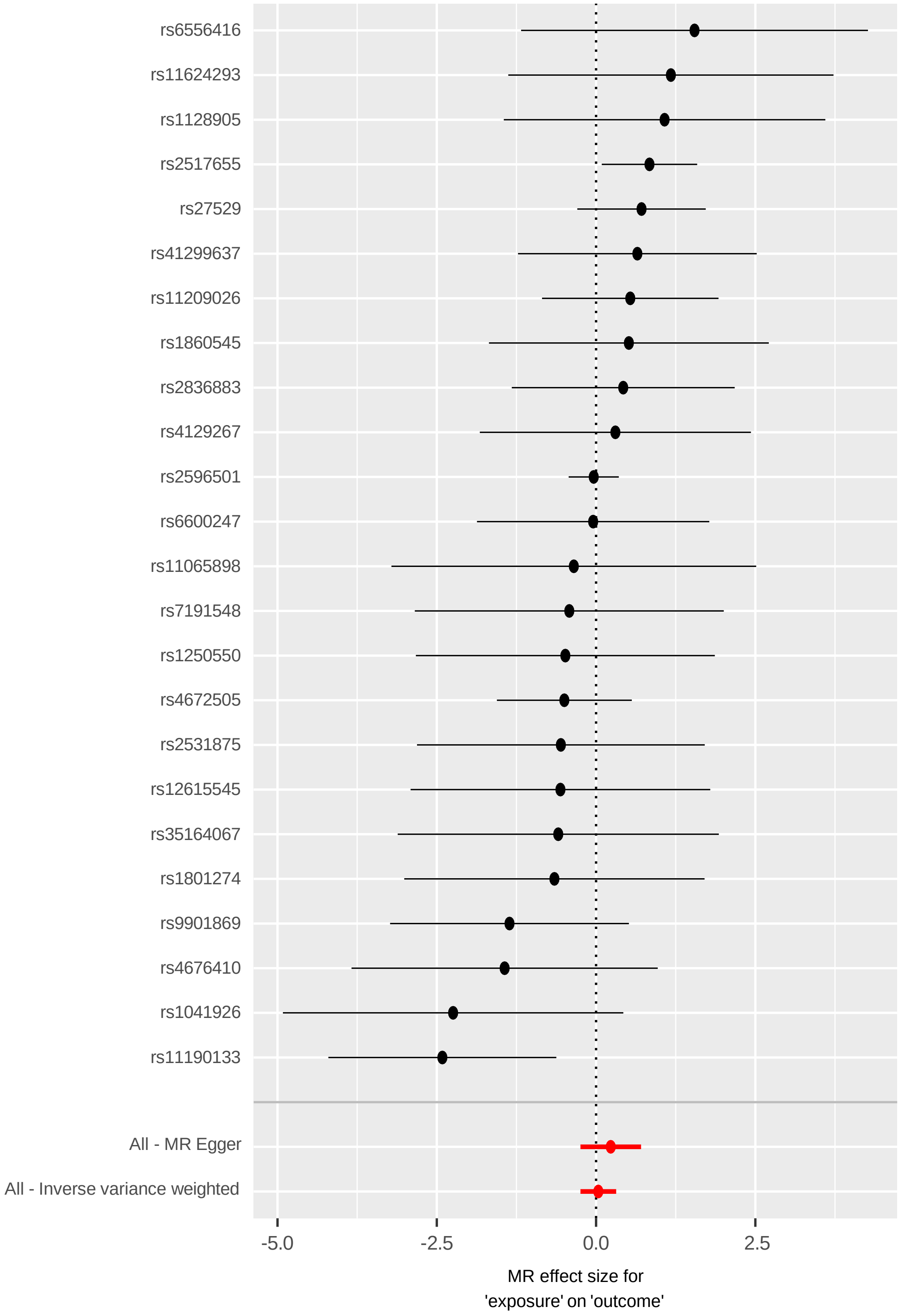

Supplementary Fig. 19 Single-SNP analysis forest plots of the effect of Ankylosing spondylitis on bronchiectasis phenotypes. Point estimates represent the variant-specific ratio estimates for each SNP (in black), and the inverse-variance weighted (IVW) estimate (in red). Horizontal lines represent 95% confidence intervals around the variant-specific ratio estimates and the

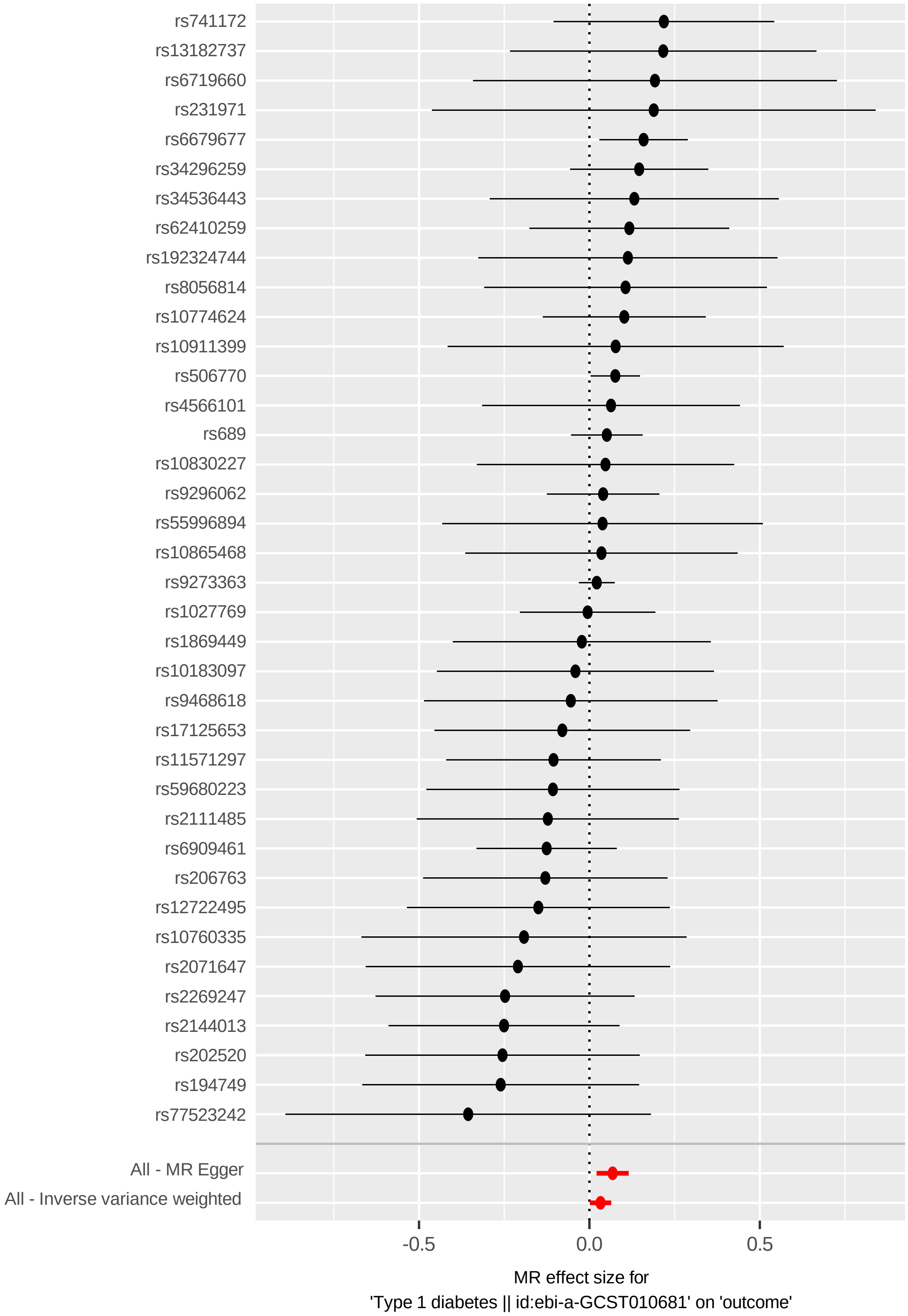

Supplementary Fig. 20 Single-SNP analysis forest plots of the effect of Type 1 diabetes on bronchiectasis phenotypes. Point estimates represent the variant-specific ratio estimates for each SNP (in black), and the inverse-variance weighted (IVW) estimate (in red). Horizontal lines represent 95% confidence intervals around the variant-specific ratio estimates and the

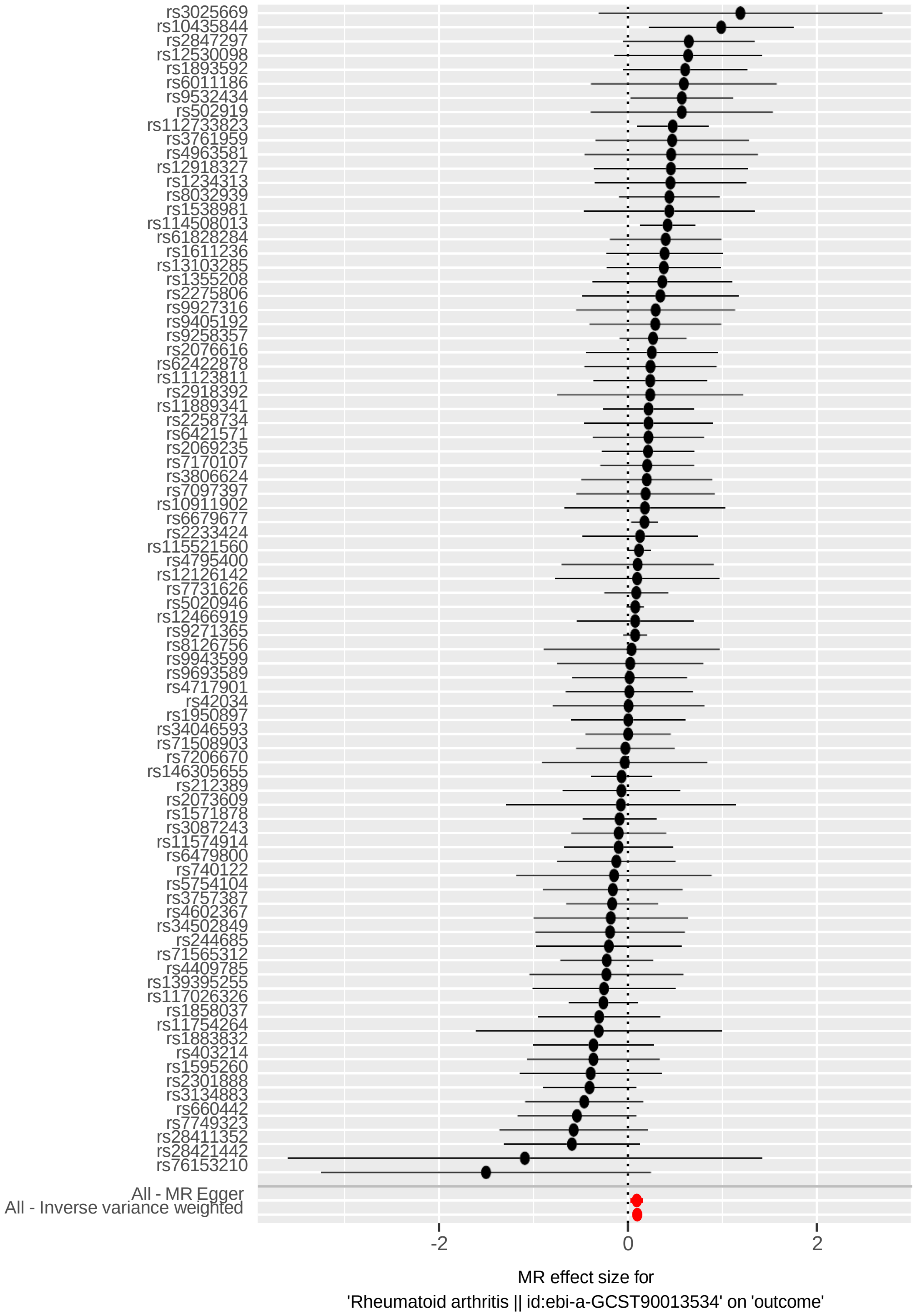

Supplementary Fig. 21 Single-SNP analysis forest plots of the effect of Rheumatoid arthritis on bronchiectasis phenotypes. Point estimates represent the variant-specific ratio estimates for each SNP (in black), and the inverse-variance weighted (IVW) estimate (in red). Horizontal lines represent 95% confidence intervals around the variant-specific ratio estimates and the

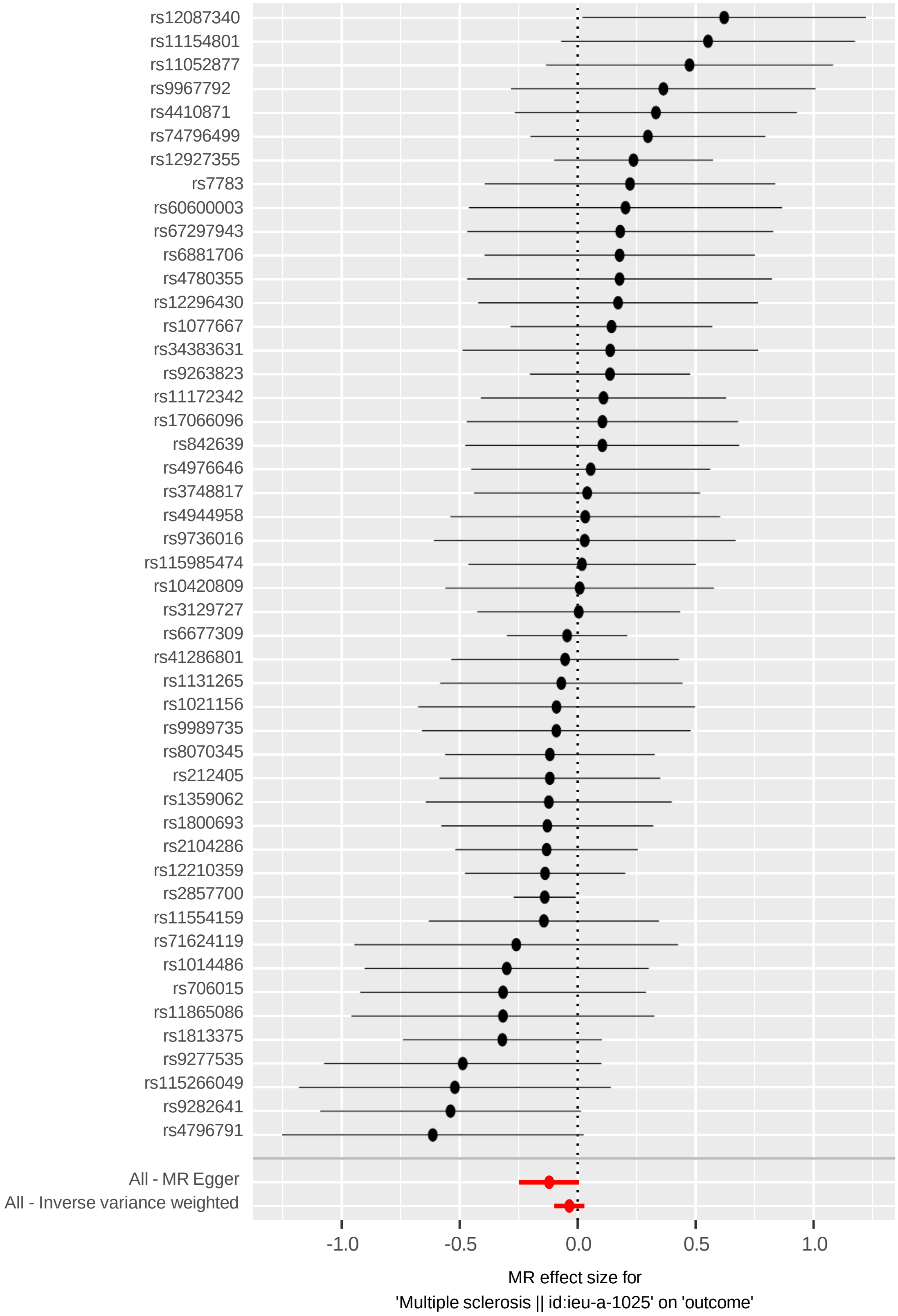

Supplementary Fig. 22 Single-SNP analysis forest plots of the effect of Multiple sclerosis on bronchiectasis phenotypes. Point estimates represent the variant-specific ratio estimates for each SNP (in black), and the inverse-variance weighted (IVW) estimate (in red). Horizontal lines represent 95% confidence intervals around the variant-specific ratio estimates and the

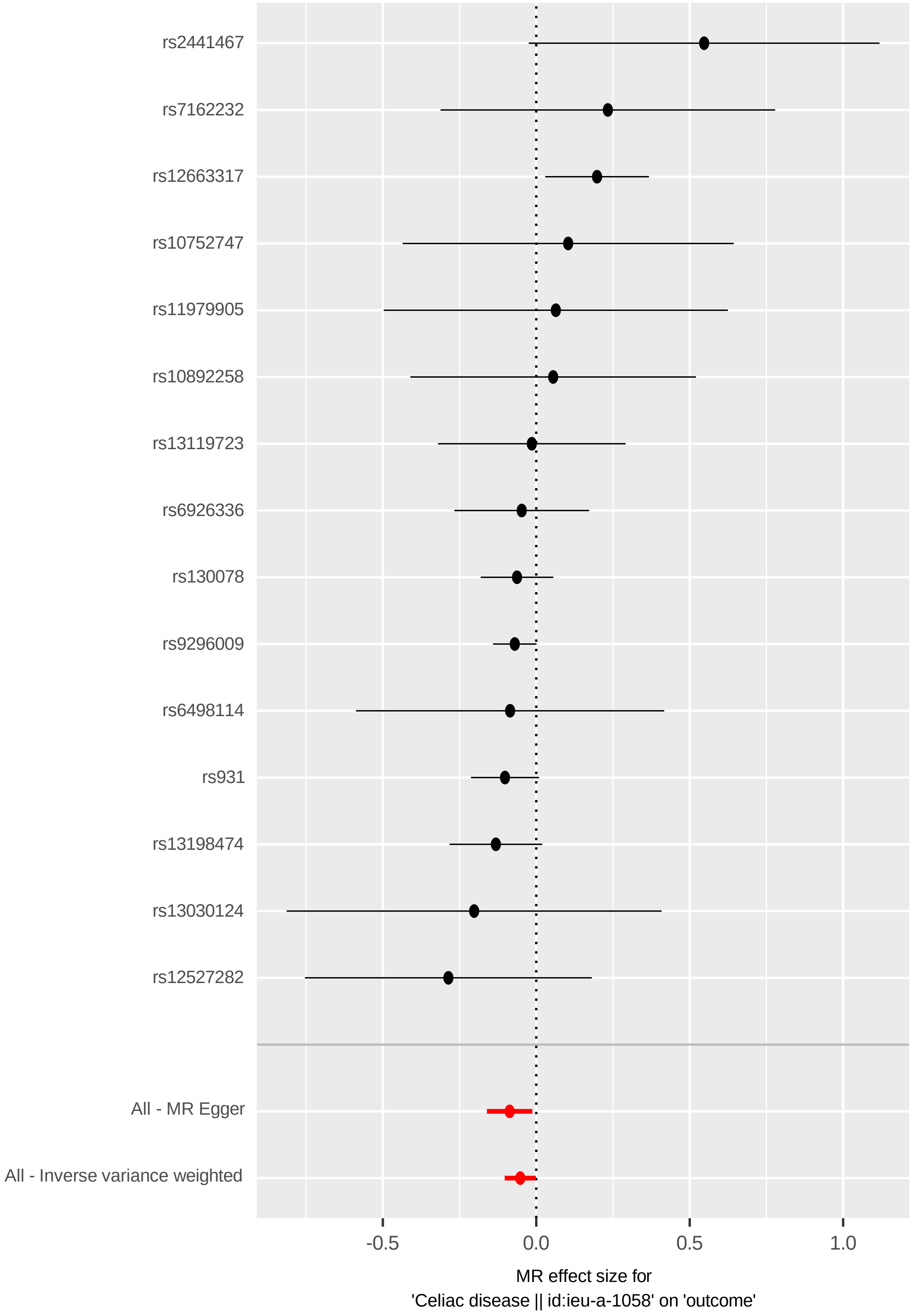

Supplementary Fig. 23 Single-SNP analysis forest plots of the effect of Celiac disease on bronchiectasis phenotypes. Point estimates represent the variant-specific ratio estimates for each SNP (in black), and the inverse-variance weighted (IVW) estimate (in red). Horizontal lines represent 95% confidence intervals around the variant-specific ratio estimates and the

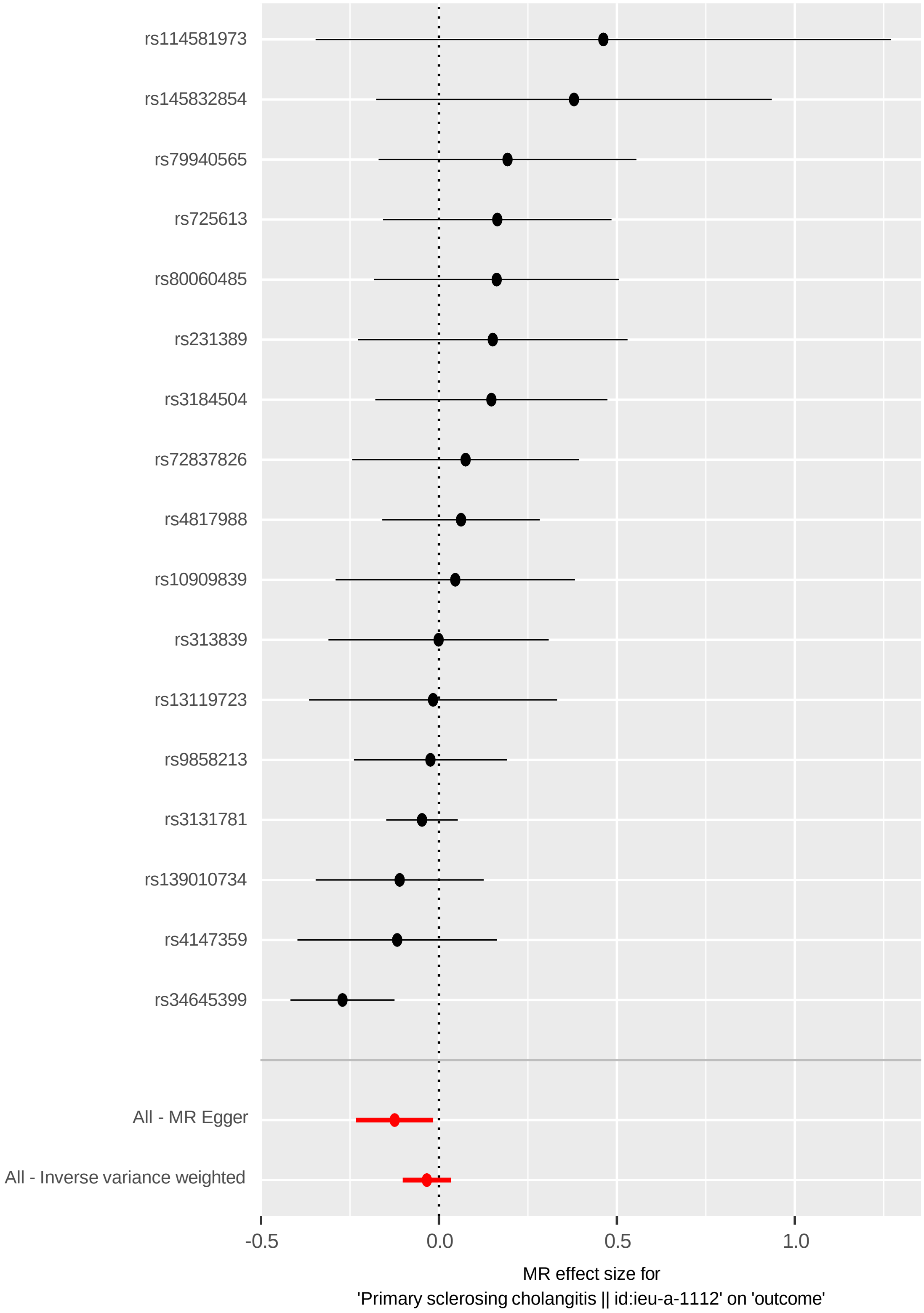

Supplementary Fig. 24 Single-SNP analysis forest plots of the effect of Primary sclerosing cholangitis on bronchiectasis phenotypes. Point estimates represent the variant-specific ratio estimates for each SNP (in black), and the inverse-variance weighted (IVW) estimate (in red). Horizontal lines represent 95% confidence intervals around the variant-specific ratio estimates and the IVW estimate

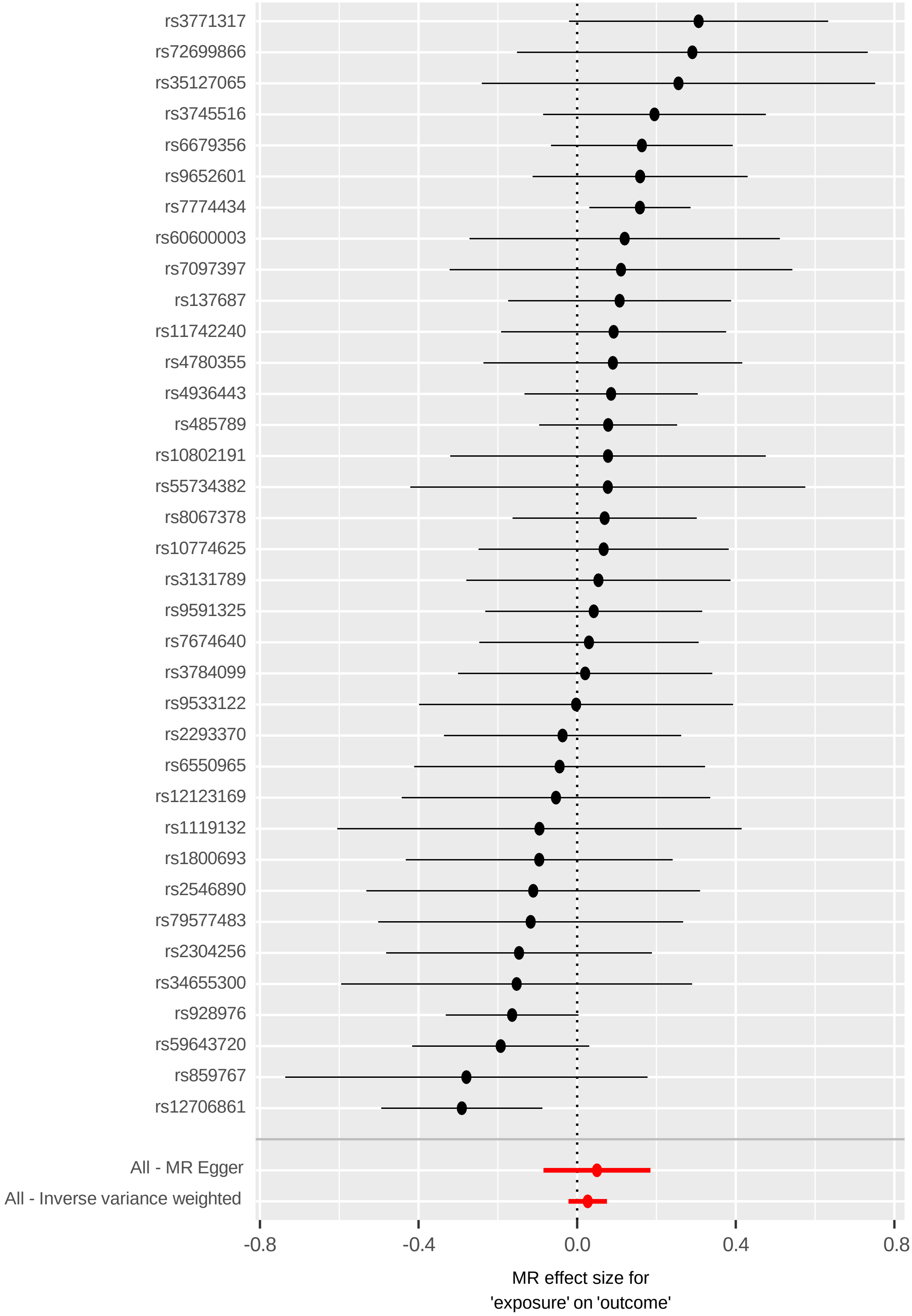

Supplementary Fig. 25 Single-SNP analysis forest plots of the effect of Primary biliary cirrhosis on bronchiectasis phenotypes. Point estimates represent the variant-specific ratio estimates for each SNP (in black), and the inverse-variance weighted (IVW) estimate (in red). Horizontal lines represent 95% confidence intervals around the variant-specific ratio estimates and the

Reference

1. de Lange, K. M. *et al.* Genome-wide association study implicates immune activation of multiple integrin genes in inflammatory bowel disease. *Nat Genet* **49**, 256–261 (2017).
2. Trynka, G. *et al.* Dense genotyping identifies and localizes multiple common and rare variant association signals in celiac disease. *Nat Genet* **43**, 1193–1201 (2011).
3. International Multiple Sclerosis Genetics Consortium (IMSGC) *et al.* Analysis of immune-related loci identifies 48 new susceptibility variants for multiple sclerosis. *Nat Genet* **45**, 1353–1360 (2013).
4. Ha, E., Bae, S.-C. & Kim, K. Large-scale meta-analysis across East Asian and European populations updated genetic architecture and variant-driven biology of rheumatoid arthritis, identifying 11 novel susceptibility loci. *Ann Rheum Dis* **80**, 558–565 (2021).
5. Bentham, J. *et al.* Genetic association analyses implicate aberrant regulation of innate and adaptive immunity genes in the pathogenesis of systemic lupus erythematosus. *Nat Genet* **47**, 1457–1464 (2015).
6. Forgetta, V. *et al.* Rare Genetic Variants of Large Effect Influence Risk of Type 1 Diabetes. *Diabetes* **69**, 784–795 (2020).
7. Tsoi, L. C. *et al.* Identification of 15 new psoriasis susceptibility loci highlights the role of innate immunity. *Nat Genet* **44**, 1341–1348 (2012).
8. Ji, S.-G. *et al.* Genome-wide association study of primary sclerosing cholangitis identifies new risk loci and quantifies the genetic relationship with inflammatory bowel disease. *Nat Genet* **49**, 269–273 (2017).
9. Cordell, H. J. *et al.* An international genome-wide meta-analysis of primary biliary cholangitis:

Novel risk loci and candidate drugs. *J Hepatol* **75**, 572–581 (2021).

10. International Genetics of Ankylosing Spondylitis Consortium (IGAS) *et al.* Identification of multiple risk variants for ankylosing spondylitis through high-density genotyping of immune-related loci. *Nat Genet* **45**, 730–738 (2013).
11. Jin, Y. *et al.* Genome-wide association studies of autoimmune vitiligo identify 23 new risk loci and highlight key pathways and regulatory variants. *Nat Genet* **48**, 1418–1424 (2016).
12. Kurki, M. I. *et al.* FinnGen provides genetic insights from a well-phenotyped isolated population. *Nature* **613**, 508–518 (2023).
13. Willer, C. J. *et al.* Discovery and refinement of loci associated with lipid levels. *Nat Genet* **45**, 1274–1283 (2013).
14. Yengo, L. *et al.* Meta-analysis of genome-wide association studies for height and body mass index in ∼700000 individuals of European ancestry. *Hum Mol Genet* **27**, 3641–3649 (2018).
15. Manousaki, D. *et al.* Genome-wide Association Study for Vitamin D Levels Reveals 69 Independent Loci. *Am J Hum Genet* **106**, 327–337 (2020).
16. Liu, M. *et al.* Association studies of up to 1.2 million individuals yield new insights into the genetic etiology of tobacco and alcohol use. *Nat Genet* **51**, 237–244 (2019).
