# Supplementary material for: Autoimmune diseases and their genetic link to bronchiectasis: insights from a genetic correlation and Mendelian randomization study
Source: Front Immunol. 2024 Apr 10;15:1343480. doi: 10.3389/fimmu.2024.1343480 (PMC11039849; doi:10.3389/fimmu.2024.1343480)
Supplement: Supplementary file 2 [file Table_1.pdf]

Table 1. Power calculations for bidirectional univariable Mendelian randomization analyses

Exposure	SNPs	Outcome	Proportion of variance in the exposure explained by the instrument (R^2)	F-statistic	Power
Crohn's disease (CD)	76	Bronchiectasis	77.97%	81.31	12%
Celiac disease (CeD)	15	Bronchiectasis	85.92%	285.82	96%
Multiple sclerosis (MS)	48	Bronchiectasis	46.59%	70.55	62%
Rheumatoid arthritis (RA)	83	Bronchiectasis	112.52%	112.26	100%
Systemic lupus erythematosus (SLE)	40	Bronchiectasis	142.93%	98.22	10%
Ulcerative colitis (UC)	56	Bronchiectasis	49.67%	69.25	44%
Type 1 diabetes (T1D)	38	Bronchiectasis	185.27%	116.39	92%
Psoriasis (PsO)	61	Bronchiectasis	1535.64%	72.29	18%
Primary sclerosing cholangitis (PSC)	17	Bronchiectasis	107.71%	130.09	39%
Primary biliary cirrhosis (PBC)	34	Bronchiectasis	87.04%	105.91	46%
Ankylosing spondylitis (AS)	24	Bronchiectasis	2.59%	127.18	5%
Vitiligo (ViT)	35	Bronchiectasis	83.83%	63.64	12%
