# Supplementary material for: Autoimmune diseases and their genetic link to bronchiectasis: insights from a genetic correlation and Mendelian randomization study
Source: Front Immunol. 2024 Apr 10;15:1343480. doi: 10.3389/fimmu.2024.1343480 (PMC11039849; doi:10.3389/fimmu.2024.1343480)
Supplement: Supplementary file 3 [file Table_2.pdf]

Table 2 Genetic Correlation Estimates for autoimmune diseases and BE by LDSC regression

Exposure	Outcome	Genetic correlation(rg)	rg (SE)	*P*
Crohn's disease (CD)	Bronchiectasis (BE)	0.220	0.105	0.037
Celiac disease (CeD)		0.331	0.314	0.292
Multiple sclerosis (MS)		0.236	0.242	0.330
Rheumatoid arthritis (RA)		0.210	0.091	0.021
Systemic lupus erythematosus (SLE)		0.166	0.162	0.304
Ulcerative colitis (UC)		0.247	0.109	0.023
Type 1 diabetes (T1D)		-0.187	0.195	0.337
Psoriasis (PsO)		0.468	0.310	0.131
Primary sclerosing cholangitis (PSC)		-0.116	0.150	0.441
Primary biliary cirrhosis (PBC)		0.288	0.178	0.106
Ankylosing spondylitis (AS)		0.596	0.370	0.107
Vitiligo (ViT)		0.380	0.478	0.426
**Phenotype**	**Heritability (h^2)**	**h^2 (SE)**	**h^2 (Z)**	**h^2 (*P*)**
Crohn's disease (CD)	33.90%	0.035	9.743	0.000
Celiac disease (CeD)	76.72%	0.316	2.430	0.015
Multiple sclerosis (MS)	132.25%	0.202	6.531	0.000
Rheumatoid arthritis (RA)	4.14%	0.008	5.495	0.000
Systemic lupus erythematosus (SLE)	49.96%	0.085	5.886	0.000
Ulcerative colitis (UC)	21.55%	0.027	7.975	0.000
Type 1 diabetes (T1D)	5.48%	0.169	0.057	2.960
Psoriasis (PsO)	57.23%	0.133	4.306	0.000
Primary sclerosing cholangitis (PSC)	36.06%	0.122	2.961	0.003
Primary biliary cirrhosis (PBC)	37.00%	0.060	6.130	0.000
Ankylosing spondylitis (AS)	232.99%	0.619	3.762	0.000
Vitiligo (ViT)	0.10%	0.001	0.925	0.355
Bronchiectasis (BE)	0.32%	0.001	2.568	0.010
