# Supplementary material for: Autoimmune diseases and their genetic link to bronchiectasis: insights from a genetic correlation and Mendelian randomization study
Source: Front Immunol. 2024 Apr 10;15:1343480. doi: 10.3389/fimmu.2024.1343480 (PMC11039849; doi:10.3389/fimmu.2024.1343480)
Supplement: Supplementary file 4 [file Table_3.pdf]

Table 3. Summary of LDSC analysis results

Genetic Correlation(rg) Result Summary

**Phenotypes**	CD	CeD	MS	RA	SLE	UC	T1D	PsO	PSC	PBC	AS	ViT	BE
Crohn's disease (CD)	NA	0.356	0.500	0.035	0.106	0.627	-0.054	0.487	0.155	0.178	1.444	0.111	0.220
Celiac disease (CeD)	0.356	NA	0.049	0.283	0.302	0.172	0.477	-0.008	0.116	0.002	0.029	0.842	0.331
Multiple sclerosis (MS)	0.500	0.049	NA	0.292	0.437	0.640	0.510	0.030	0.677	0.978	0.204	0.289	0.236
Rheumatoid arthritis (RA)	0.035	0.283	0.292	NA	0.391	0.146	0.309	0.170	0.108	0.276	0.381	-0.212	0.210
Systemic lupus erythematosus (SLE)	0.106	0.302	0.437	0.391	NA	0.220	0.387	-0.008	0.282	0.331	0.200	0.218	0.166
Ulcerative colitis (UC)	0.627	0.172	0.640	0.146	0.220	NA	0.110	0.438	0.480	0.254	1.426	-0.050	0.247
Type 1 diabetes (T1D)	-0.054	0.477	0.510	0.309	0.387	0.110	NA	0.025	0.490	0.187	-0.264	-0.059	-0.187
Psoriasis (PsO)	0.487	-0.008	0.030	0.170	-0.008	0.438	0.025	NA	-0.051	0.330	0.141	-0.637	0.468
Primary sclerosing cholangitis (PSC)	0.155	0.116	0.677	0.108	0.282	0.480	0.490	-0.051	NA	0.164	0.277	0.096	-0.116
Primary biliary cirrhosis (PBC)	0.178	0.002	0.978	0.276	0.331	0.254	0.187	0.330	0.164	NA	0.561	-0.357	0.288
Ankylosing spondylitis (AS)	1.444	0.029	0.204	0.381	0.200	1.426	-0.264	0.141	0.277	0.561	NA	-0.629	0.596
Vitiligo (ViT)	0.111	0.842	0.289	-0.212	0.218	-0.050	-0.059	-0.637	0.096	-0.357	-0.629	NA	0.380
Bronchiectasis (BE)	0.220	0.331	0.236	0.210	0.166	0.247	-0.187	0.468	-0.116	0.288	0.596	0.380	NA

Genetic Correlation(rg) Pval Result Summary

**Phenotypes**	CD	CeD	MS	RA	SLE	UC	T1D	PsO	PSC	PBC	AS	ViT	BE
Crohn's disease (CD)	NA	0.023	0.016	0.467	0.086	3.60E-17	0.458	0.025	0.026	0.010	4.09E-07	0.532	0.037
Celiac disease (CeD)	0.023	NA	0.453	0.196	0.162	0.323	0.063	0.932	0.728	0.993	0.772	0.106	0.292
Multiple sclerosis (MS)	0.016	0.453	NA	0.138	0.038	0.002	0.121	0.724	0.004	0.006	0.048	0.447	0.330
Rheumatoid arthritis (RA)	0.467	0.196	0.138	NA	9.53E-10	0.006	1.17E-04	0.392	0.061	7.65E-06	0.053	0.210	0.021
Systemic lupus erythematosus (SLE)	0.086	0.162	0.038	9.53E-10	NA	7.38E-05	0.033	0.971	0.079	0.002	0.440	0.368	0.304
Ulcerative colitis (UC)	3.60E-17	0.323	0.002	0.006	7.38E-05	NA	0.153	0.067	3.88E-06	1.80E-04	6.33E-08	0.786	0.023
Type 1 diabetes (T1D)	0.458	0.063	0.121	1.17E-04	0.033	0.153	NA	0.931	0.036	0.173	0.441	0.859	0.337
Psoriasis (PsO)	0.025	0.932	0.724	0.392	0.971	0.067	0.931	NA	0.849	0.298	0.278	0.276	0.131
Primary sclerosing cholangitis (PSC)	0.026	0.728	0.004	0.061	0.079	3.88E-06	0.036	0.849	NA	0.152	0.296	0.710	0.441
Primary biliary cirrhosis (PBC)	0.010	0.993	0.006	7.65E-06	0.002	1.80E-04	0.173	0.298	0.152	NA	0.131	0.173	0.106
Ankylosing spondylitis (AS)	4.09E-07	0.772	0.048	0.053	0.440	6.33E-08	0.441	0.278	0.296	0.131	NA	0.249	0.107
Vitiligo (ViT)	0.532	0.106	0.447	0.210	0.368	0.786	0.859	0.276	0.710	0.173	0.249	NA	0.426
Bronchiectasis (BE)	0.037	0.292	0.330	0.021	0.304	0.023	0.337	0.131	0.441	0.106	0.107	0.426	NA
